# Winter all year round in urgent and emergency care: a large retrospective analysis of routinely collected NHS data across England, 2021–2022

**DOI:** 10.1186/s12913-026-14253-3

**Published:** 2026-03-04

**Authors:** Jen Lewis, Richard M. Jacques, Madina Hasan, Susan Croft, Richard Campbell, Rebecca Simpson, Simone Croft, Sophie Williams, Charles Gutteridge, Suzy Gallier, Felicity Evison, Elizabeth Sapey, Amy Dillon, Rachel Denholm, Erik Mayer, Quinta Davies, Jo Knight, Vishnu Chandrabalan, Michael George, Thomas Phillips, Matt Stammers, Suzanne Mason

**Affiliations:** 1 School of Medicine and Population Health, University of Sheffield, Sheffield, UK; 2 Data Connect, University of Sheffield, Sheffield, UK; 3 Sheffield Teaching Hospitals NHS Foundation Trust, Sheffield, UK; 4 Barts Life Sciences, Barts Health NHS Trust, London, UK; 5 Clinical Informatics, Barts Health NHS Trust, London, UK; 6 NIHR Midlands Patient Safety Research Collaboration and NIHR Biomedical Research Centre, University Hospitals Birmingham NHS Foundation Trust, Birmingham, UK; 7 PIONEER Data Hub in Acute Care, School of Medical Sciences, University of Birmingham, Birmingham, UK; 8 Bristol Medical School, University of Bristol, Bristol, UK; 9 Bristol NIHR Biomedical Research Centre, Bristol, UK; 10 Health Data Research UK South West, Bristol, UK; 11 iCARE Secure Data Environment, Digital Collaboration Space, St Mary’s Hospital, Imperial College Healthcare NHS Trust & Imperial College London, London, UK; 12 Faculty of Medicine, Department of Surgery and Cancer, Imperial College London, London, UK; 13 Department of Data Science, Lancashire Teaching Hospitals NHS Trust, Preston, UK; 14 Lancaster Medical School, Lancaster University, Bailrigg, Lancaster, UK; 15 Southampton Emerging Therapies and Technologies (SETT) Centre, Southampton, UK; 16 University Hospital Southampton Foundation Trust (UHSFT), Southampton, UK; 17 University of Southampton, Southampton, UK

**Keywords:** Winter pressures, Urgent care, Emergency care, NHS, Avoidable admission, Emergency department, Routine data

## Abstract

**Background:**

‘Winter pressures’ in urgent and emergency care (UEC) are widely accepted but have had little empirical attention. Amidst annually increasing demand for UEC and reports of extreme strain during winter, we aimed to understand the extent and nature of seasonal demand by analysing routine data from Emergency Departments (ED) and acute Admitted Patient Care (APC) episodes across England.

**Methods:**

This was a retrospective observational analysis using data from 26 hospitals and 22 EDs between 2021-11-1 and 2022-10-31 comparing emergency attendances and acute admissions between winter (October-March) and summer (April-September). Main outcomes included ED waiting times, length of admissions, the number of investigations, treatments and procedures received, and whether the contact was considered avoidable. Using a novel ‘federated’ approach exploiting local relationships with data providers, regional researchers analysed local data and provided summary statistics and analysis results to the lead site. Aggregation of summary results established a picture of seasonal demand across the country, and an understanding of regional variation in seasonal trends.

**Results:**

1,549,205 ED attendances (775,810 winter; 50.1%) and 747,685 APC admissions (368,910 winter, 49.3%) were analysed. We found no systematic seasonal differences in the number or nature of presentations. While regional variation existed for many outcomes, no nationally consistent effect of winter was found for any measure.

**Conclusions:**

Winter pressures in UEC may not be driven by large differences in the number, avoidability or acuity of ED attendances or APC admissions. Rather, UEC may be operating at or near to capacity all year, meaning small fluctuations in demand or in the complexity of presentations may cause significant strain on an over-burdened system. Focus on managing seasonal demand should be modified to address year-round pressure. Effective policy may require structural reconfiguration to better regulate demand.

**Supplementary Information:**

The online version contains supplementary material available at 10.1186/s12913-026-14253-3.

## Background

Emergency departments in UK hospitals are reported, year-on-year, to experience increasing pressures during winter months [1]. Given this, there continues to be a focus on relieving the impact of these ‘winter pressures’ [2], and it is often assumed that there will be a large spike in demand for emergency care during winter [3]. However, little research has been conducted that evidences this assumed peak in demand, and, particularly in more recent years, there has been speculation around whether this winter peak now truly exists or whether high demand is simply now experienced all year. In 2016 the Health Foundation and Nuffield Trust reported that “Pressure normally seen during the winter months is increasingly visible at other times of the year” [4], and in 2018 it was documented that ‘Winter all year round’ was becoming an increasingly common descriptor [5].

One recent study based in North-West England showed no increases in acute hospital admissions but a slight increase in ED attendances in winter 2018–2019 [6], and some evidence exists that the number of ED attendances may, in fact, *decrease* in winter, while the proportion of ED attendances resulting in admission may increase [7]. However, a recent audit suggests that acute care performance is poorer in winter than summer, that presenting acuity is generally more severe, and that the NHS believes that both ED attendances and acute admissions increase during winter [8, 9].

Given the shortage of peer-reviewed research in this area and the lack of consistency in the publications that do exist, we aimed to describe and quantify seasonal fluctuation in demand, pressure and patient demographic and clinical characteristics in EDs and acute hospitals across England over a recent, post-covid year. This was achieved by examining seasonal trends in routine data and documenting demographics, clinical characteristics, and key performance measures regarding adult ED attendances and unplanned acute hospital admissions from various hospitals across England.

## Methods

This was a retrospective, observational study utilising routinely collected NHS healthcare data and was a collaboration of the HDRUK Regional Linked Data Consortium [10]. We used a ‘federated’ approach to the analysis of routine data collected by multiple NHS acute trusts across England. We use ‘federated’ to describe a process whereby record-level data from regional NHS acute trusts pertaining to their managed hospitals was accessed and analysed by local research centres within the consortium. Resulting summary statistics and frequencies were subsequently aggregated to provide a national picture without requiring a single centre to access record-level data from all regions.

The study was co-ordinated by a lead research centre (Sheffield), who supplied a data specification to all other centres. The data specification was aligned with the nationally mandated Commissioning Data Set 6.2 Type 011: Emergency Care (ECDS) [11] and nationally mandated Commissioning Data Set 6.2 Type 130 Admitted Patient Care – Finished General Episodes (APC) [12]. These datasets contain data regarding ED attendances and acute admissions, respectively, from hospitals managed by NHS acute trusts. These datasets are primarily collected for administrative purposes but are commonly used in UK health services research. The lead centre (Sheffield) also supplied a data analysis plan and template results tables with each research centre (Additional File 1). Each research centre established local ethics, data sharing and data acquisition processes with their corresponding NHS trusts. The lead centre aggregated results following regional analysis.

### Record inclusion criteria

We used total population sampling in that all eligible ED attendances and acute admissions from NHS Acute Trusts linked with consortium members within the specified time frame were included. For ECDS data, a record was eligible if it pertained to a first unplanned adult (age 18+) emergency care attendance for a new clinical condition or deterioration of a chronic condition at a Type 1 ED between 2021-11-1 and 2022-10-31. Planned and unplanned follow-up attendances within 7 days and related to the initial attendance were not eligible. A type 1 ED is a 24-hour consultant-led service with full resuscitation facilities and designated accommodation for the reception of emergency care patients [13]. We excluded data from Type 2, 3 and 4 EDs.

For APC data, a record was eligible if it pertained to the first episode in an unplanned emergency hospital inpatient spell in a general hospital with acute care provision, between 2021-11-1 and 2022-10-31.

### Variables

Variables in Table 1 were summarised overall and individually for each ED/hospital. For continuous variables, the total N, mean, standard deviation, median, range and upper and lower quartiles were reported for winter months and non-winter (summer) months. For categorical variables, appropriate categories and their associated codes within the dataset (Additional File 2 & 3) were pre-specified, and for each category, the number and percent were reported. In accordance with data-sharing agreements and to ensure confidentiality and anonymity, rounding to nearest 5 and *n* < 10 low number suppression was applied.

**Table 1:** Variables included in the aggregated analysis

	ED attendances	Acute admissions
**Demographic variables**		
Categorical	Patient age	Patient age
	Patient gender	Patient gender
	Patient ethnicity	Patient ethnicity
	Patient deprivation (Townsend Quintile)	Patient deprivation (Townsend Quintile)
	Comorbidities (y/n)	Comorbidities (y/n)
	Care home residence (y/n)	Care home residence (y/n)
**Key pressure indicators**		
Continuous	ED waiting time (mins: arrival to assessment)	Length of stay (nights)
	ED total time (mins: arrival to conclusion)	
	N urgent investigations	
	N urgent treatments	
Categorical	Overall urgency	Potentially avoidable admission (y/n)
		Operative procedures (y/n)
		Length of stay ( > = 2 nights)
**Clinical characteristics**		
Categorical	Seasonal diagnosis	Seasonal diagnosis
	Mode of arrival	Source of admission
	Source of referral	Discharge destination
	Time of attendance	Time of admission
	Attendance acuity	
	Chief complaint	
	Discharge destination	

### Winter

‘Winter’ was considered to consist of the six months between October 1st and March 31st, in line with previous work [14]. In a sensitivity analysis we also explored the data using the more publicly accepted definition of winter of the three months between December 1st and February 28th.

### Urgency

We considered an ED attendance to be non-urgent where it met the criteria outlined in O’Keeffe et al. [15], defined when a patient received:

1. No or only ‘non-urgent’ investigations (urinalysis, pregnancy test, dental investigation);
2. No or only ‘non-urgent’ treatments (prescriptions, written advice, dental treatment);
3. A non-urgent discharge status (streamed from ED to GP following initial assessment, discharged, or left ED before assessment or treatment).

This was designed to indicate cases where suitable care could have reasonably been provided outside of the urgent care environment. All other treatments and investigations were considered urgent, and all attendances meeting these criteria were deemed urgent.

### Time of attendance/admission

‘In-hours’ was defined as 8am-6pm Monday to Friday. All other times were considered ‘out of hours’.

### Other categorical variables

We examined the number of contacts that received a primary diagnosis that was considered to be seasonal. Codes meeting these criteria are listed in Additional File 2. Chief complaint was reduced into a smaller number of categories, which are listed in Additional File 3. We considered potentially avoidable admissions to be those where the primary diagnosis was an ‘Ambulatory Emergency Care Condition’ (AECC). These conditions identify patients who could reasonably be assessed, diagnosed and treated on the same day, avoiding admission. Emergency admissions for AECCs were identified using codes recorded in the primary diagnosis field for the first episode in the inpatient spell. These are listed in Additional File 4. Seasonal diagnoses, chief complaint categories and AECCs were agreed by clinical authors (SuC & SM). All other encoding for categorical variables is included in Additional File 2.

### Regression analysis

Key pressure indicators were also analysed by multivariable linear or logistic regression to explore whether key demographic or clinical characteristics may obscure any impact of winter in descriptive summaries.

For ED attendances, indicators included ED time to assessment, ED total time, urgency of attendance, and whether < 2 or ≥ 2 treatments and investigations were received. For acute admissions, indicators included the length of stay categorised as < 2 or ≥ 2 nights, whether the admission was potentially avoidable, and whether any operative procedures were received. All regressions used winter as the primary (binary) predictor and included the following covariates: Age (continuous); gender; ethnicity; Townsend deprivation quintile; care home residence; presence of a seasonal diagnosis; and time of contact (all categorical).

### Aggregation

Researchers at the lead site aggregated summary data to give a national and regional picture of seasonal variation. Results were visualised by site, and pooled statistics - weighted according to the sample size at each site - were calculated for each variable to provide overall summary results. For categorical variables, pooled percentages for each category were calculated, and for continuous variables, pooled means, standard deviations, medians and interquartile ranges were calculated. Results from regression analyses were visualised using forest plots.

### Subgroup & sensitivity analyses

We performed a stratified analysis to examine whether particular subgroups of ED attendants or inpatients demonstrated particular effects of winter. We stratified ED attendances by arrival mode (arrival by ambulance or other), urgency of the attendance (urgent and non-urgent) and time of the attendance (In-hours and out-of-hours). For acute admissions, we stratified according to the discharge destination (care home, further medical care, or other), whether the attendance was considered potentially avoidable, and the time of admission (in-hours and out-of-hours).

We also performed a sensitivity analysis by re-examining all variables, comparing only a three-month winter (December-February) with a nine-month non-winter season.

## Results

We received results from 13 trusts, comprising data from 38 hospitals. Of these, 24 had an associated ED. Twelve hospitals were specialist and thus did not meet the inclusion criteria; these were excluded from the aggregated analysis. The final aggregated analysis included data from 26 Acute inpatient hospitals and 22 Type 1 Emergency Departments across London, Birmingham, Lancaster, Bristol, Southampton, and South Yorkshire.

Findings were extensive, so we focus only on patient demographics and key pressure indicators in the main paper. Additional results in line with Table 1, including clinical characteristics of ED attendances and acute admissions, are available in Additional File 5.

### Total attendances and admissions

**Fig. 1:**
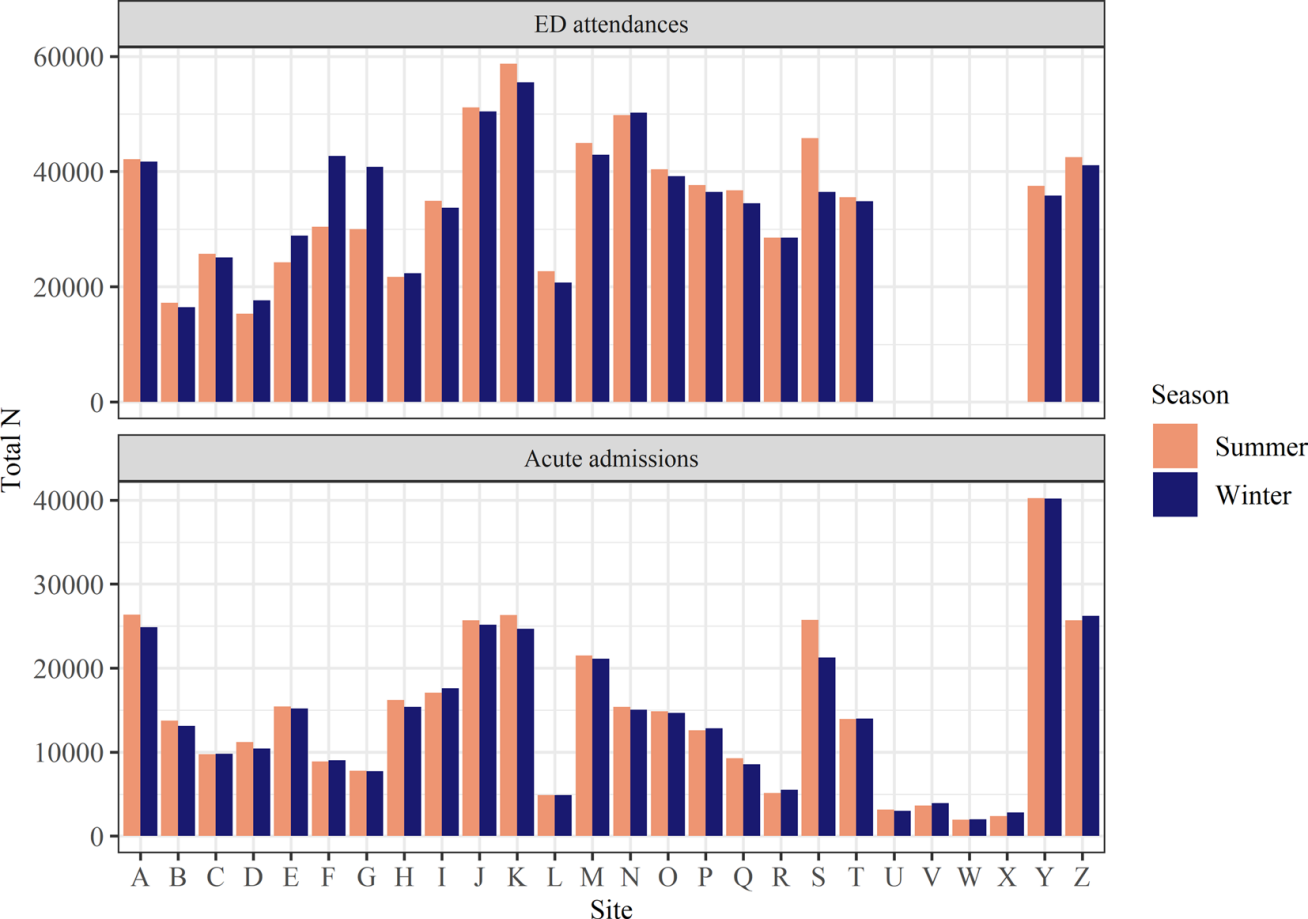
Total ED attendances & acute admissions by site

Sites did not always report missing values for a given variable, so no definitive total number of attendances or admissions was available. The estimated total for each site is presented in Table 2, taken as the maximum rounded N across demographic variables for each site, and displayed in Fig. 1. Overall, there were approximately 1,549,205 ED attendances and 747,685 acute admissions analysed. There were approximately 2,415 more ED attendances in winter, but approximately 9,845 more acute admissions in summer. In percentage terms these differences were small (~ 1%), and there was no clear seasonal pattern across sites. Two sites (F, G) saw considerably more ED attendances in winter, but this was not seen for acute admissions and was not seen for any other site. Some sites saw more attendances in the summer months, particularly site S.

**Table 2:** N & percent of attendances and admissions by site

Site	ED attendances			Acute Admissions		
	Winter (%)	Summer (%)	Overall	Winter (%)	Summer (%)	Overall
A	41,730 (49.8)	42,135 (50.2)	83,865	24,870 (48.5)	26,380 (51.5)	51,250
B	16,470 (48.9)	17,235 (51.1)	33,705	13,140 (48.9)	13,725 (51.1)	26,865
C	25,075 (49.4)	25,680 (50.6)	50,755	9,815 (50.1)	9,760 (49.9)	19,575
D	17,620 (53.6)	15,280 (46.4)	32,900	10,445 (48.2)	11,205 (51.8)	21,650
E	28,865 (54.4)	24,240 (45.7)	53,105	15,180 (49.6)	15,445 (50.4)	30,625
F	42,720 (58.4)	30,415 (41.6)	73,135	9,030 (50.4)	8,900 (49.6)	17,930
G	40,770 (57.6)	29,995 (42.4)	70,765	7,725 (49.9)	7,765 (50.1)	15,490
H	22,325 (50.7)	21,710 (49.3)	44,035	15,360 (48.7)	16,190 (51.3)	31,550
I	33,705 (49.1)	34,900 (50.9)	68,605	17,580 (50.7)	17,065 (49.3)	34,645
J	50,405 (49.6)	51,135 (50.4)	101,540	25,170 (49.5)	25,710 (50.5)	50,880
K	55,500 (48.6)	58,695 (51.4)	114,195	24,685 (48.4)	26,305 (51.6)	50,990
L	20,705 (47.7)	22,675 (52.3)	43,380	4,865 (49.9)	4,895 (50.2)	9,760
M	42,925 (48.8)	44,970 (51.2)	87,895	21,105 (49.5)	21,510 (50.5)	42,615
N	50,210 (50.2)	49,760 (49.8)	99,970	15,045 (49.4)	15,395 (50.6)	30,440
O	39,195 (49.3)	40,395 (50.8)	79,590	14,680 (49.7)	14,840 (50.3)	29,520
P	36,450 (49.2)	37,650 (50.8)	74,100	12,845 (50.5)	12,585 (49.5)	25,430
Q	34,465 (48.4)	36,730 (51.6)	71,195	8,535 (47.9)	9,280 (52.1)	17,815
R	28,485 (50.0)	28,480 (50.0)	56,965	5,490 (51.6)	5,145 (48.4)	10,635
S	36,450 (44.3)	45,755 (55.7)	82,205	21,255 (45.3)	25,720 (54.8)	46,975
T	34,825 (49.5)	35,560 (50.5)	70,385	13,980 (50.1)	13,930 (49.9)	27,910
U	No ED	No ED	No ED	2,995 (48.7)	3,150 (51.3)	6,145
V	No ED	No ED	No ED	3,915 (51.9)	3,625 (48.1)	7,540
W	No ED	No ED	No ED	2,000 (50.6)	1,955 (49.4)	3,955
X	No ED	No ED	No ED	2,805 (54.4)	2,355 (45.6)	5,160
Y	35,820 (48.83)	37,530 (51.2)	73,350	40,170 (50.0)	40,255 (50.1)	80,425
Z	41,095 (49.18)	42,470 (50.8)	83,565	26,225 (50.5)	25,685 (49.5)	51,910
Total	775,810 (50.1)	773,395 (49.9)	1,549,205	368,910 (49.3)	378,775 (50.7)	747,685

**Fig. 2:**
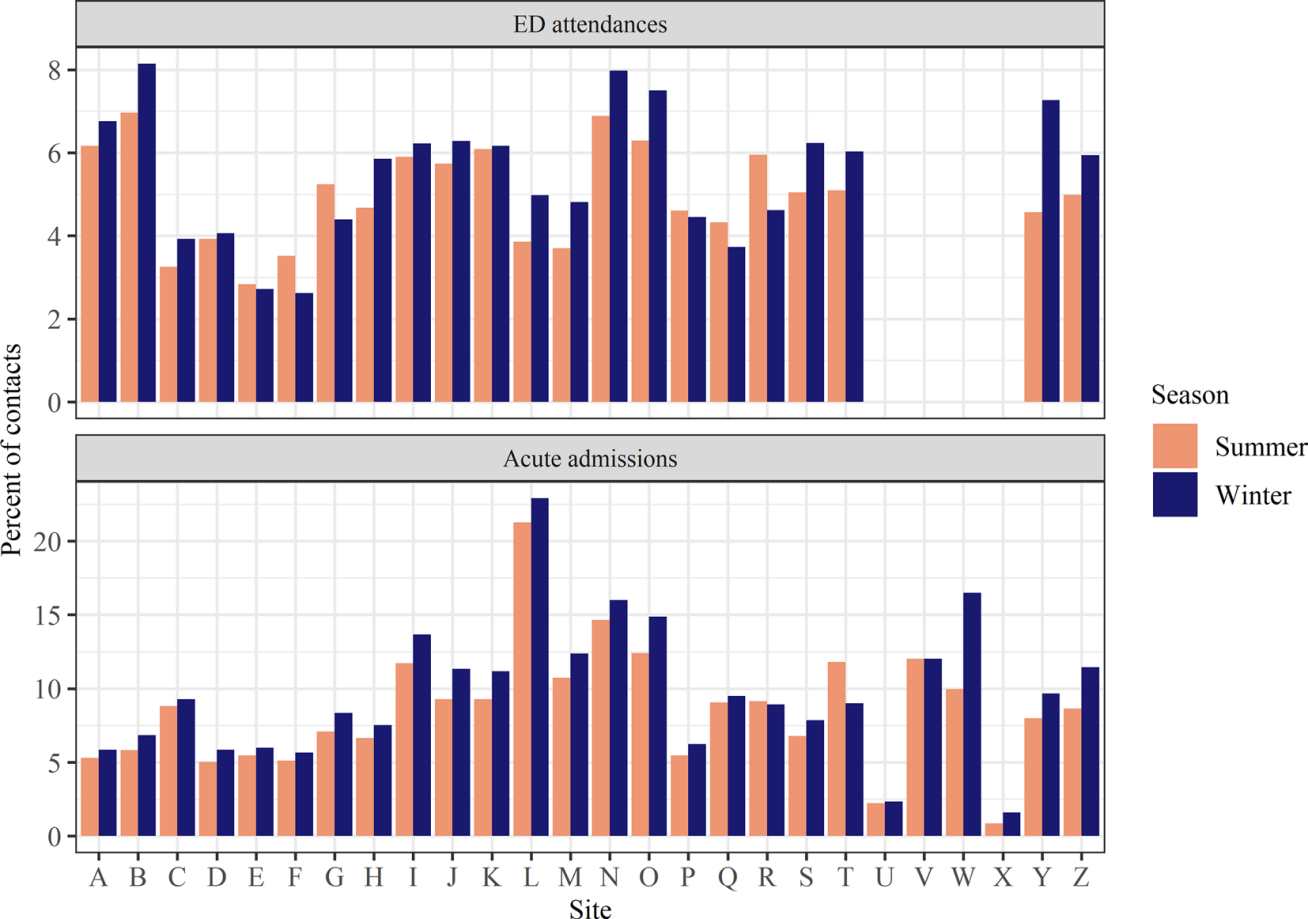
Prevalence of seasonal diagnoses among ED attendances & acute admissions by site

The percentage of ED attendances and acute admissions with a winter-related diagnosis is shown in Fig. 2. Overall, 9.8% of acute admissions in winter included a primary seasonal diagnosis. Summer months showed a marginal reduction, at 8.5% of admissions. This represents a difference of approximately 3,960 patients across all sites. In ED, 5.6% of attendees in winter had a seasonal diagnosis, compared with 5.1% in summer months, a difference of approximately 4,000 more patients with a winter-related diagnosis in winter overall.

### Demographics

We examined patient age, gender, ethnicity, deprivation quintile, care home residence and presence of comorbidities. Tables 3 and 4 show weighted, pooled percentages across sites, and the range of percentages across sites for each variable. Percentages for each variable by site are shown in A5-1.1 to A5-1.6 Figures in Additional File 5. While patient demographics varied between hospitals, differences between winter and summer were within a single percentage point for almost all variables. For ED attendances, there was a slight seasonal percentage difference in patients with comorbidities; unexpectedly this indicated more patients with comorbidities attending in the summer months, though this was not shown in all sites (A5-1.5 Figure; Additional File 5).

**Table 3:** Categorical demographics for ED attendances

	October-March		April-September		Overall	
	%	min %-max %	%	min %-max %	%	min %-max %
Age						
< 20	3.65	2.37–6.70	3.29	2.40–4.68	3.47	2.42–5.68
20–24	9.07	6.11–14.47	8.74	6.12–13.22	8.91	6.11–13.84
25–29	8.95	6.11–12.05	8.85	6.59–12.26	8.90	6.35–12.15
30–34	8.78	6.56–11.27	8.76	6.52–11.12	8.77	6.67–11.18
35–39	7.87	6.00–9.95	7.93	6.29–9.66	7.90	6.14–9.81
40–44	7.04	5.69–9.38	7.04	5.89–9.13	7.04	5.83–9.25
45–49	6.46	5.57–7.85	6.43	5.60–7.66	6.45	5.63–7.75
50–54	6.96	5.59–7.69	6.89	5.63–7.57	6.92	5.61–7.53
55–59	6.62	5.53–7.47	6.73	5.82–7.37	6.67	5.68–7.35
60–64	5.90	4.55–6.90	6.07	4.88–6.70	5.99	4.72–6.80
65–69	5.14	3.76–6.04	5.28	4.09–6.02	5.21	3.93–5.92
70–74	5.46	3.89–7.37	5.52	3.86–7.23	5.49	4.01–7.30
75–79	5.63	3.23–8.15	5.90	3.49–8.52	5.76	3.36–8.34
80–84	5.18	3.05–7.88	5.32	3.23–7.53	5.25	3.14–7.71
85+	7.30	3.59–10.88	7.26	3.74–11.15	7.28	3.93–11.02
Ethnicity						
Asian/ Asian British	12.64	0.48–36.60	12.95	0.42–37.39	12.80	0.45–37.00
Black/ Black British	5.81	0.39–17.12	5.68	0.29–17.84	5.74	0.33–17.48
Mixed	1.57	0.24–2.67	1.61	0.27–2.68	1.59	0.25–2.66
White	60.20	27.90–93.62	60.74	27.95–92.76	60.47	27.93–93.18
Other	6.24	0.00–39.09	5.23	0.00–36.46	5.74	0.00–38.00
Unknown/ missing	13.53	4.07–33.04	13.79	4.59–34.13	13.66	4.34–33.59
Gender						
Female	52.22	48.79–56.02	51.87	48.51–55.12	52.05	48.80–55.16
Male	47.77	43.98–51.21	48.11	44.88–51.49	47.94	44.84–51.20
Unknown/ missing	0.01	0.00–0.06	0.01	0.00–0.06	0.01	0.00–0.06
Deprivation						
Q1 (Least)	8.48	0.18–33.71	8.89	0.15–33.51	8.68	0.16–33.61
Q2	10.28	0.44–22.78	10.73	0.46–23.22	10.51	0.45–23.01
Q3	13.55	1.39–23.19	14.15	1.51–23.63	13.85	1.50–23.41
Q4	20.41	3.90–33.07	20.71	3.58–33.05	20.56	3.74–33.06
Q5 (Most)	40.58	5.51–93.14	39.75	7.19–93.71	40.16	6.37–93.43
Unknown/ missing	6.70	0.00–30.37	5.77	0.00–31.46	6.23	0.00–30.88
Comorbidities						
No	60.16	2.19–92.72	55.29	7.45–87.25	57.77	4.89–88.48
Yes	39.84	7.28–97.81	44.71	12.75–92.55	42.23	11.52–95.11
Care home residence						
No	81.42	0.00–99.75	84.23	0.00–99.76	82.82	0.00–99.76
Yes	4.37	0.00–38.53	3.44	0.00–40.11	3.91	0.00–39.19
Unknown/ missing	14.21	0.00–100.00	12.33	0.00–100.00	13.28	0.00–100.00

Data are presented as pooled percentages, with a min-max percentage range across sites. Presence of comorbidities was reported by 13, and care home residence by 17 of the 22 sites

**Table 4:** Categorical demographics for acute admissions

	Winter		Summer		Overall	
	%	min %-max %	%	min %-max %	%	min %-max %
Age						
< 20	1.57	0.51–3.75	1.59	0.00–2.81	1.58	0.29–3.29
20–24	4.44	1.43–9.50	4.56	1.53–9.72	4.50	1.54–9.61
25–29	5.24	2.37–8.75	5.32	2.04–8.95	5.28	2.20–8.85
30–34	5.63	2.57–7.55	5.78	2.25–9.21	5.70	2.41–8.34
35–39	5.48	2.57–7.13	5.56	2.66–7.67	5.52	2.61–7.33
40–44	5.20	2.06–7.38	5.34	2.66–6.70	5.28	2.36–6.96
45–49	5.23	2.78–6.95	5.30	3.78–7.45	5.26	3.28–7.18
50–54	6.40	5.04–9.27	6.28	4.19–10.00	6.34	4.61–9.63
55–59	6.86	5.24–11.41	6.89	5.41–10.43	6.88	5.48–10.96
60–64	7.15	5.75–11.09	7.28	6.23–11.43	7.22	6.19–11.27
65–69	7.13	5.75–11.60	7.20	5.96–10.43	7.17	6.07–11.06
70–74	8.60	6.04–12.15	8.31	5.88–10.64	8.45	6.16–11.41
75–79	9.28	6.12–14.51	9.38	6.16–13.93	9.33	6.14–14.10
80–84	8.86	6.00–13.48	8.77	5.63–14.71	8.81	5.82–14.10
85+	12.92	4.37–23.25	12.44	5.63–23.70	12.68	5.06–23.48
Ethnicity						
Asian/ Asian British	10.83	0.31–32.91	11.19	0.40–34.01	11.01	0.36–33.44
Black/ Black British	4.48	0.00–17.20	4.47	0.20–17.69	4.48	0.10–17.36
Mixed	1.08	0.17–2.30	1.11	0.25–2.50	1.09	0.21–2.40
White	64.39	29.35–94.01	63.91	29.47–93.16	64.15	29.41–93.48
Other	3.68	0.00–26.44	3.64	0.00–26.81	3.66	0.00–26.63
Unknown/ missing	15.54	3.70–30.42	15.67	3.87–29.14	15.61	3.78–29.78
Gender						
Female	53.08	39.71–59.73	53.02	34.26–59.51	53.05	37.22–59.62
Male	46.92	40.27–60.29	46.98	40.49–65.74	46.95	40.38–62.78
Deprivation						
Q1 (Least)	11.66	0.26–40.46	11.52	0.13–41.42	11.59	0.21–40.92
Q2	13.19	0.65–23.66	12.92	0.69–22.67	13.06	0.67–22.93
Q3	15.18	1.77–23.87	15.30	1.48–24.27	15.24	1.63–24.07
Q4	21.68	3.45–32.75	21.81	2.77–33.12	21.75	3.12–32.94
Q5 (Most)	33.46	6.31–93.66	33.35	6.54–94.76	33.41	6.56–94.19
Unknown/ missing	4.82	0.00–31.69	5.10	0.00–32.41	4.96	0.00–32.06
Comorbidities						
No	7.19	0.00–16.22	7.41	0.00–17.91	7.30	0.00–16.28
Yes	92.81	83.78–100.00	92.59	82.09–100.00	92.70	83.72–100.00
Care home residence						
No	99.19	93.98–100.00	99.22	93.63–100.00	99.21	93.81–100.00
Yes	0.44	0.00–2.01	0.39	0.00–1.74	0.42	0.00–1.79
Unknown/ missing	0.37	0.00–6.02	0.39	0.00–6.37	0.38	0.00–6.19

Data are presented as pooled percentages, with a min-max percentage range across sites. Presence of comorbidities was reported by 18, and care home residence by 17 of the 26 sites

### Key pressure indicators

#### ED attendance key pressure indicators

Tables 5 and 6 show aggregated results for continuous and categorical measures. Sites P, Q and R were excluded from the aggregation of time to assessment, and site H excluded from both waiting time measures, due to high levels of data errors for these outcomes including high frequencies of impossible values. Sites T and Z were excluded from the aggregation of investigations, treatments and urgency due to errors in categorising.

**Table 5:** Pooled mean & SD and median & IQR for continuous outcomes for ED attendances

Variable	Season	*N*	Pooled Mean (Pooled SD)		Pooled Median [Pooled IQR]	
Total time in ED (minutes)	October-March	753,820	364.32	(437.46)	264	[158–483]
	April-September	751,470	362.17	(337.82)	279	[159–500]
Time to assessment (minutes)	October-March	640,585	100.86	(178.34)	94	[22–168]
	April-September	632,535	107.37	(119.47)	90	[24–185]
Urgent investigations (per attendance)	October-March	699,825	3.88	(3.49)	4	[1–7]
	April-September	695,300	3.80	(3.46)	4	[0–5]
Urgent treatments (per attendance)	October-March	699,830	1.42	(1.88)	1	[0–2]
	April-September	695,300	1.43	(1.90)	1	[0–2]

**Table 6:** Pooled percentage of urgent and non-urgent attendances, with min-max range across sites

	October-March		April-September		Overall	
	%	min %-max %	%	min %-max %	%	min %-max %
Urgency						
Non-urgent	16.55	4.37–39.81	18.07	4.59–47.43	17.31	4.48–43.67
Urgent	83.45	60.19–95.63	81.93	52.57–95.41	82.69	56.33–95.52

There was little seasonal difference in the number of urgent investigations and treatments received in ED. Total time in ED varied in opposite directions for mean and median measures, where mean time was 2 min longer in winter, but median time 15 min longer in summer (Table 5). This suggests a more skewed distribution of total time in winter. In summer months, waits may be typically slightly closer to the average. The reverse pattern was found for time to assessment. There was a tendency for more ED attendances to be urgent in winter, though this difference was slight.

### ED waiting time

**Fig. 3:**
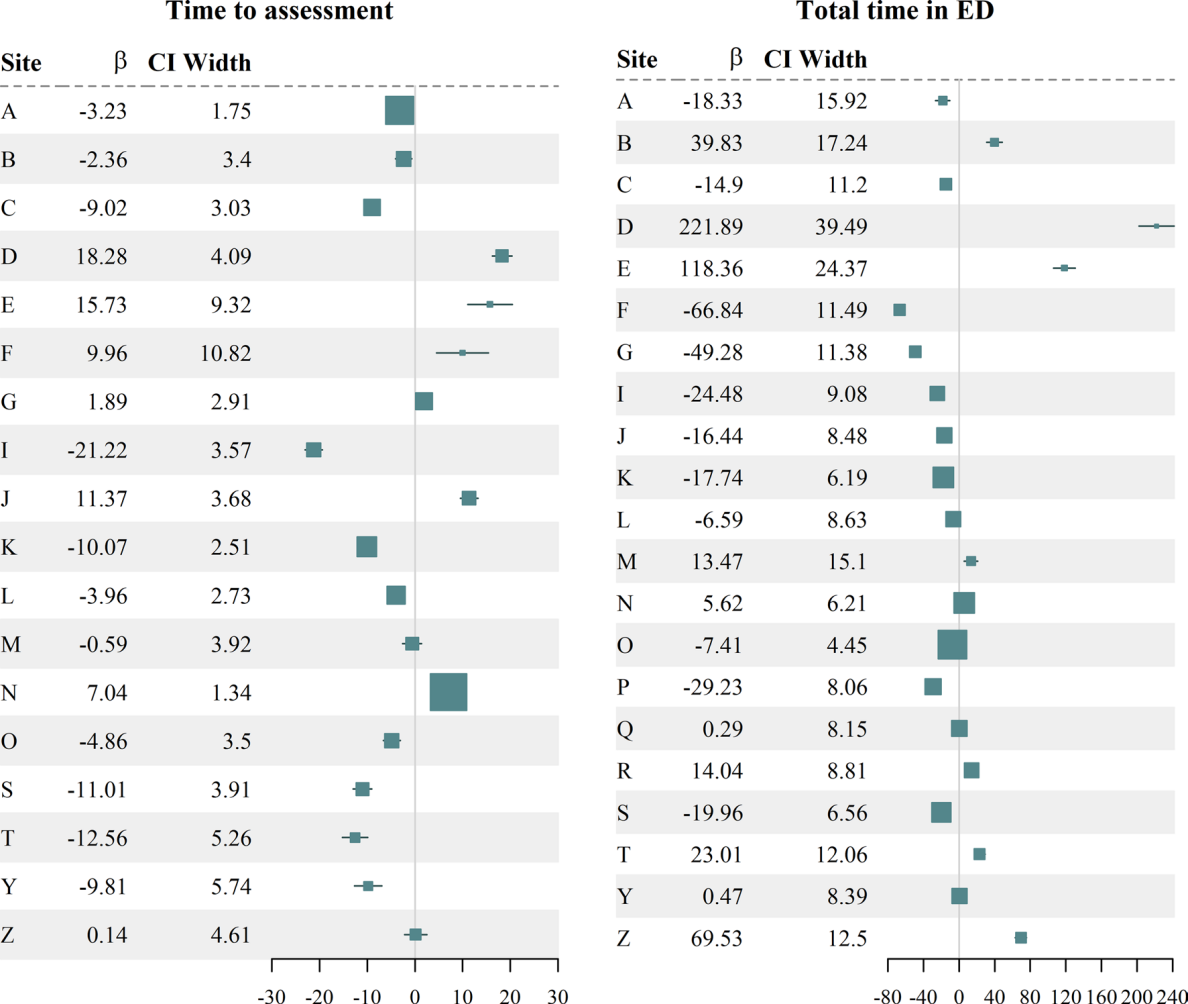
Effect of winter on ED waiting times. Forest plots show coefficient estimates for the adjusted effect of winter on time to assessment and total time in ED in minutes and their 95% confidence intervals. Box size is based on the certainty of the estimate

Crude seasonal differences in ED waiting times were inconsistent across sites (A5-2.1 Figure; Additional File 5). To explore the impact of ED waiting time whilst controlling for key confounders, sites performed adjusted linear regressions on ED waiting time. Results of these regressions are aggregated in Fig. 3. These are consistent with crude differences showing no clear overall impact of winter.

**Fig. 4:**
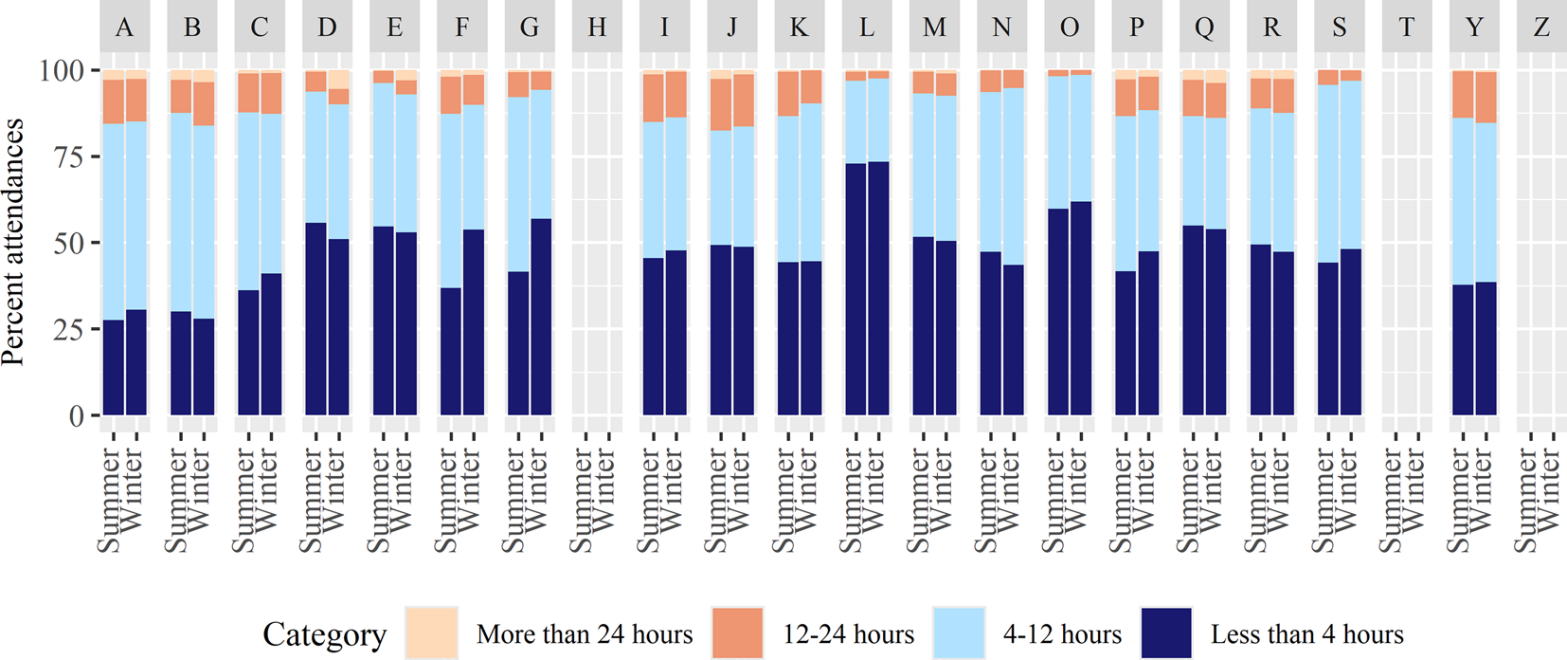
Percentage of ED attendances concluded within 4 h, 12 h, and 24 h of arrival

To investigate further, we examined the percentage of cases being seen before the key 4-hour target and those seen within 12 and 24 h (Fig. 4; missing data for sites H, T and Z). Again, results vary regionally, but there was no evidence suggesting longer waiting times overall in winter. Indeed, proportionally more attendances in winter were seen in less than 4 h (48.3%), compared to summer (46.2%), and rates of attendances waiting more than 12 h were similar across seasons (10.1% in winter; 10.7% in summer).

### Urgency of ED attendances

**Fig. 5:**
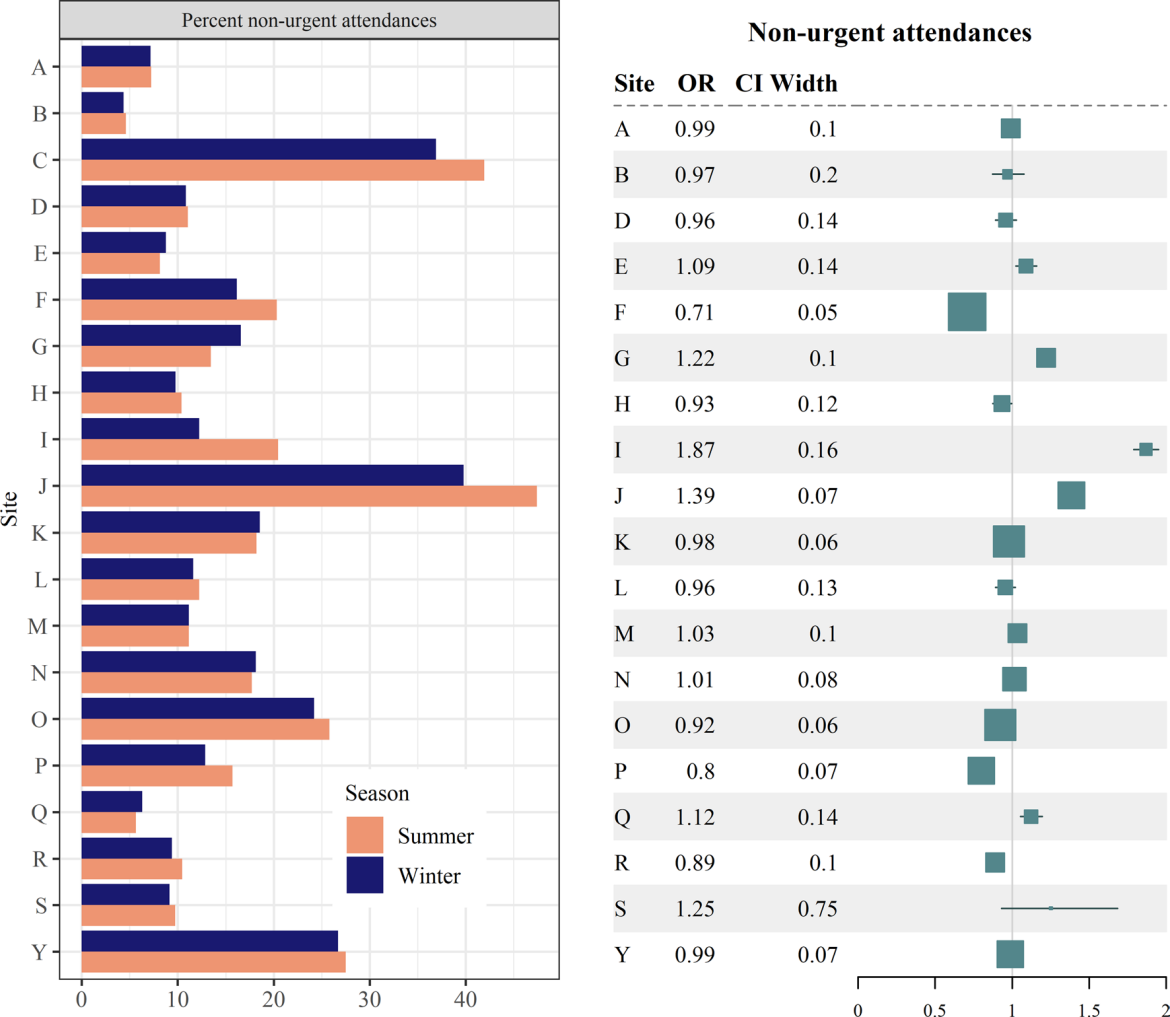
Non-urgent attendances. (Left) Percent of non-urgent attendances by site & season. (Right) Forest plot of odds ratio (OR) estimates for the adjusted effect of winter on the likelihood of an ED attendance being non-urgent

The small tendency for higher overall levels of urgency in winter (Table 6) was variable across sites (Fig. 5, left). Adjusted logistic regressions (Fig. 5, right; missing data for site C) indicate that this effect may not be robust when accounting for patient characteristics, as the effect appears to be attenuated or even reversed for some sites after adjustment (sites I, J). However, there was little consistency overall, and most sites showed no significant effect.

### Acute admissions key pressure indicators

Table 7 shows aggregated results for admissions outcomes. Site T did not report length of stay (LOS) as a continuous variable. Sites I, J & K were excluded from the aggregation of the number of procedures due to data errors. Pooled estimates of LOS suggest slightly longer stays during winter, though this difference was small. An examination of LOS as a continuous outcome showed the median LOS was identical across seasons for most sites (A5-2.4 Figure; Additional File 5). Summary data suggests no seasonal difference in the likelihood of a patient receiving one or more procedures.

**Table 7:** Pooled percentages for categorical outcomes for acute admissions, with min-max range across sites

	October-March		April-September		Overall	
	%	min %-max %	%	min %-max %	%	min %-max %
Length of stay						
2 or more nights	49.43	28.14–85.10	48.77	27.98–83.82	49.1	28.21–84.45
Less than 2 nights	50.57	14.9–71.86	51.23	16.18–72.02	50.9	15.55–71.79
Procedures						
None	36.77	0.00–71.34	36.96	0.00–67.76	36.87	0.00–69.510
1 or more	44.82	25.41–83.01	44.93	24.26–84.29	44.88	25.25–83.59
Unknown/ missing	18.41	0.00–74.59	18.10	0.00–75.74	18.26	0.00–74.75
Admission type						
Potentially avoidable	41.64	18.7–61.48	41.14	22.25–60.63	41.39	20.45–60.74
Non-avoidable	58.36	38.52–81.3	58.86	39.37–77.75	58.61	39.26–79.55

### Length of stay & procedures received

**Fig. 6:**
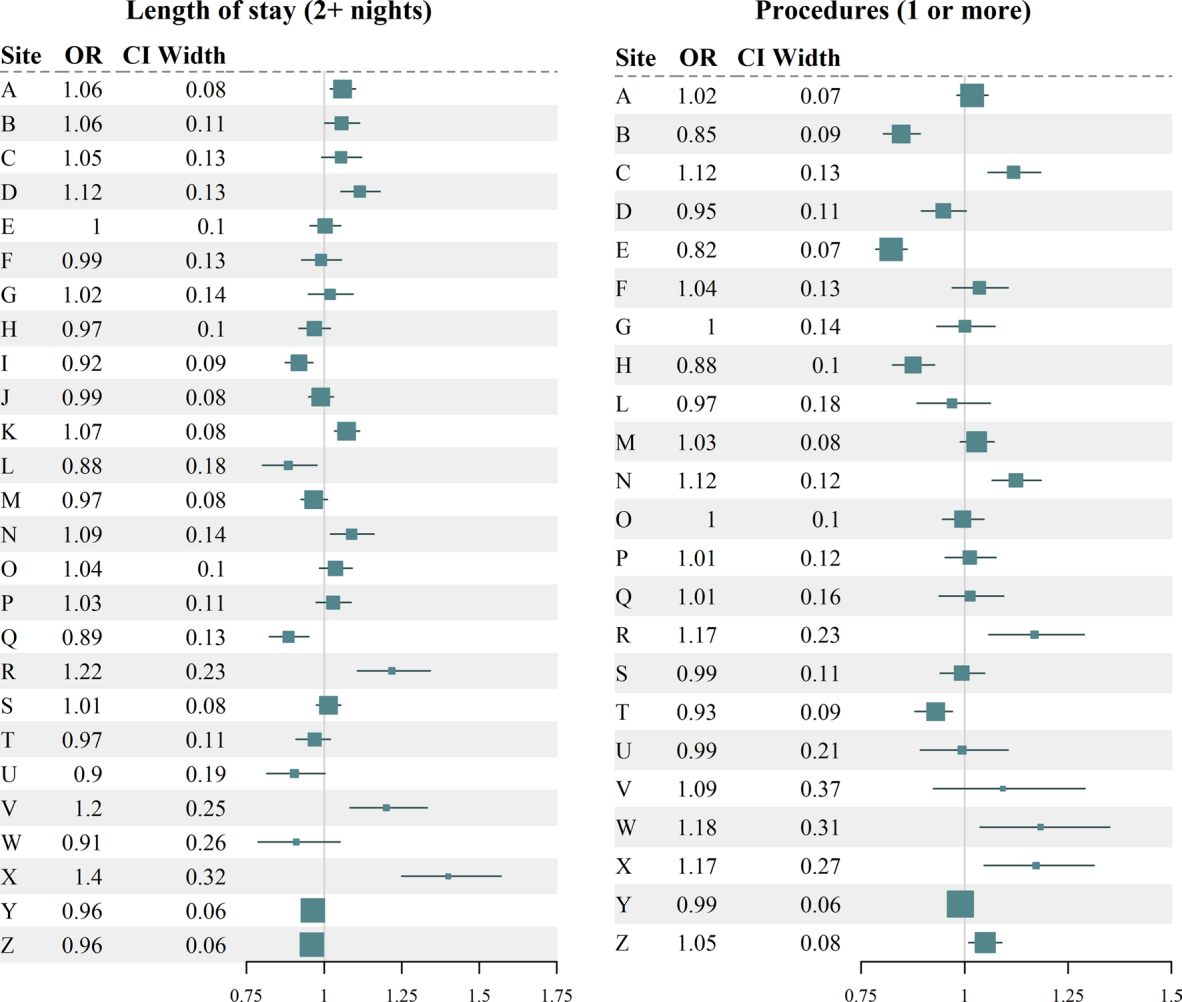
Length of stay and precedures. Forest plots of odds ratio (OR) estimates for the adjusted effect of winter on the likelihood of an acute admission having a length of stay of 2 or more nights (left), and the likelihood of an acute admission requiring at least one operative procedure (right)

Adjusted odds ratios for the impact of winter on a LOS of 2 or more nights and at least one procedure are shown in Fig. 6. For both measures, some sites showed a positive or negative effect of winter, but in many cases no effect was observed.

#### Potentially avoidable admissions

**Fig. 7:**
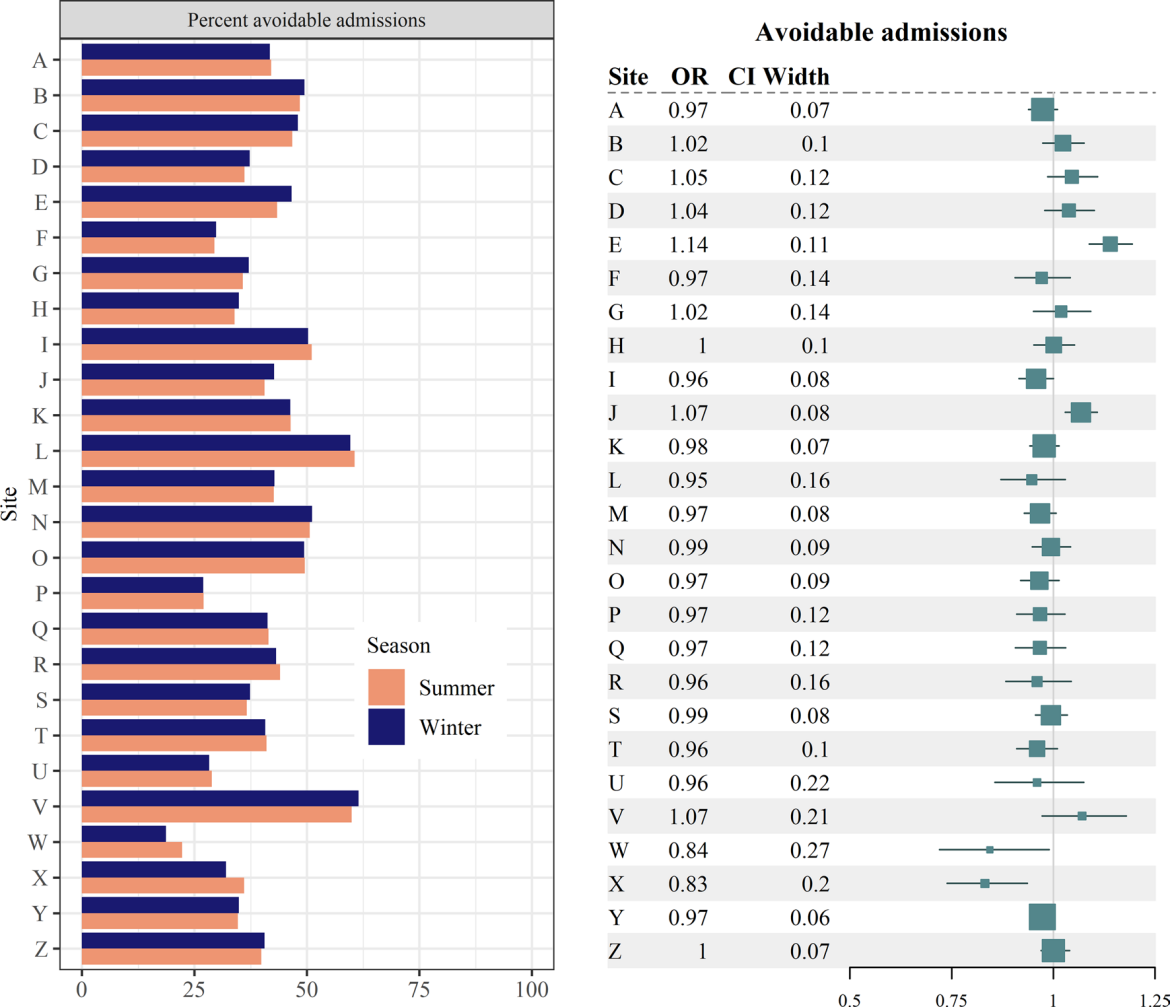
Potentially avoidable admissions (Left) Percent of potentially avoidable admissions by site & season. (Right) Forest plot of odds ratio (OR) estimates for the adjusted effect of winter the likelihood of an acute admission being considered potentially avoidable

Pooled estimates of the percent of potentially avoidable admissions showed no clear seasonal difference (Table 7). The percent of potentially avoidable admissions by season is shown in Fig. 7 (left). Adjusted odds ratios (Fig. 7, right) were consistent with the unadjusted percentages.

### Subgroup & sensitivity analyses results

After stratifying by key demographic and clinical features, differences between strata levels were observed (e.g., in-hours ED attendances typically had shorter waiting times than out-of-hours), but we did not observe interactions between strata and seasonality. This suggests that the lack of effect of winter is robust and not an artefact of broad-level analysis obscuring results in specific subgroups. Results of the sensitivity analysis examining a 3-month winter were in line with the primary analysis and did not reveal any further effects of winter (results were lengthy and are not shown for brevity but are available from the corresponding author upon request).

## Discussion

This federated approach to analysis of routine emergency care data from 26 English hospitals has not revealed any systematic differences in either the number or the characteristics of adult ED attendances and acute admissions between winter and summer months. This is perhaps surprising, as it is inconsistent with commonly reported significant increases in demand during winter. However, it is in line with recent suggestions that demand is equivalent to ‘winter all year round’ [5].

The lack of a systematic effect of winter in our sample was robust, without any measure we summarised showing any clear or consistent impact. This was the case for all key pressure indicators, clinical characteristics and demographic variables, regardless of the 6-month or 3-month winter definition, and when measures were stratified by key characteristics. Even amongst widely utilised key pressure indicators, such as ED waiting time, which are regularly anticipated to increase in winter, we did not observe marked differences in the summary measures provided, with some sites even showing minor improvements during the winter months.

Demand for emergency care in recent years has increased overall - not only seasonally. Reasons for this have not been entirely elucidated but are likely to include factors understood to drive non-urgent attendance, such as difficulty accessing primary care [16], the convenience of a 24-hour service [17] and overly cautious triaging by non-clinical NHS 111 call handlers who may send patients to ED who do not require emergency care [18]. Urgent care use also appears to be growing with increasing rates of referrals for urgent cancer checks [19]. In addition, evidence shows high rates of attrition and early retirement among ED staff [20], and delayed discharge resulting from poorly organised and communicated care in the community for medically fit but frail patients [21]. Studies have also shown inconsistent implementations of services such as Same Day Emergency Care [22] and GP services within ED settings [23]. Together, these many factors may combine to create a context in which services are effectively at capacity regardless of season.

Despite an ongoing NHS focus on reducing winter pressure [1, 9, 24, 25], we are not aware of any large structural initiatives that may have reduced or diverted winter demand; rather response to and mitigation of pressures varies significantly across hospitals [9] and specific approaches left to local hospitals and Trusts [25]. Some measures of increased demand in winter may be obscured by these existing, local seasonal mitigation strategies – for example, the temporary repurposing of Same Day Emergency Care (SDEC) resources as additional inpatient beds, increased hospital beds and clinical staff [9], cancellations of elective surgeries and outpatient appointments [26] and even by turning patients away if capacity has been reached [27]. Other measures may not be visible in this routine data, for instance, variation in ambulance waiting times before patients are checked in to ED: if ED is broadly at capacity year-round, additional pressure might be revealed in increased ambulance queues in winter. However, this study does allow us to draw some inferences regarding these questions. For instance, the overall number of attendances and admissions was consistent across seasons: repurposing of alternative facilities would be expected to result in an increase in these. Secondly, if more patients were turned away in winter due to capacity being reached, we would expect to see a difference in the demographics, clinical characteristics and general acuity of patients across seasons, but we did not see differences in any of these measures. Additional ambulance arrivals resulting in longer queues might be expected to result in higher arrivals by ambulance in winter, but this was not seen.

Increased ambulance waits might be reflected in increased handover delay in winter, rather than the number of ambulance arrivals. This might point to the notion of poorer flow through UEC in winter, rather than increased demand per se. In this study we have primarily examined demand, while it may be that winter pressures are more related to problems with flow. For instance, respiratory viruses might complicate internal bed moves or discharges, restricting flow. However, some of the measures we examined would be expected to reflect such problems, including ED wait times, length of stay, and the number of investigations, procedures and discharges. We did not see such patterns. However, flow is a complex notion that is likely to be specialty-specific [28] and examination of flow into and out of certain wards might reveal aspects of seasonal pressure that we have not found.

### Impact of COVID-19

The study year is less than two years on from the onset of the COVID-19 pandemic, and at this time, the transmission of the disease was primarily driven by pandemic dynamics, masking any inherent seasonality expected of established, endemic respiratory coronaviruses [29]. This could contribute to the masking of a seasonal pattern in pressures on UEC. We included COVID-19 in our definition of seasonal diagnoses; attendances and admissions for respiratory infections including COVID-19 accounted for a small proportion of contacts overall (Tables A5-4.1 & A5-4.2). It is unlikely that the lack of seasonality in early COVID-19 transmission would mask winter pressures to the extent we have seen in this study, though it may be a contributing factor that should be explored in more recent data.

### Implications

In contrast with our findings, reports of additional strain in emergency care during the winter months are common. It is possible that the small percentage increase in numbers of ED attendances in winter, equating to around 2,415 more ED attendances across the 22 EDs included in the study, may be sufficient to cause significant strain that itself is not quantifiable using routine data. Similarly, the approximately 4,000 additional seasonal diagnoses across EDs and acute wards may represent an additional degree of complexity in presentations that current systems find hard to cope with. In this case, however, our findings still suggest that urgent care systems typically operate at an unacceptably high capacity if small seasonal fluctuations in percentage terms are sufficient to cause significant strain. This, however, cannot reconcile the notion of winter pressures with the nearly 10,000 additional acute admissions that were seen in summer compared to winter.

Our findings suggest that seasonal mitigation measures aimed at managing fluctuating demand across the year may no longer be an effective approach within the emergency care setting. Rather, measures typically deployed seasonally to reduce and manage this demand may now be necessary all year. This finding supports the recent Darzi review [30], indicating the problem is more fundamental than just a seasonal one and emphasising the need for some restructuring or reprovisioning of urgent care services, such that there is better triage and signposting of patients to appropriate sources of care. In the case of potentially avoidable attendances and admissions, the community provision must be robust and effective, reducing the need to attend ED [30].

Demand for - and patient outcomes following - emergency care often remain consistent after reconfigurations of emergency healthcare services and even closures of EDs [31, 32]. Conversely, the expansion of EDs and the introduction of other urgent care services aimed at reducing demand in ED, do not tend to improve patient flow and may even increase demand [33]. Such measures include NHS 111, a 24-hour telephone and online triaging service for urgent but non-emergency health concerns [33]; and walk-in centres which are services for minor but potentially urgent healthcare concerns [34]. Measures known to reduce demand at EDs, such as increasing primary care provision [35] or increasing the level of clinical advice available through NHS 111 [18] are advisable, but often not in the power of those responsible for managing patient flow through ED and into acute wards. Going forward, strategies to manage high year-round demand should take a ‘best-of-both’ approach, utilising strategies known to help mitigate pressure during previously seasonal periods of high demand and through structural reconfiguration where possible to reduce demand.

### Strengths & limitations

This study is one of the first to use large-scale routine data to examine the impact of winter on key performance measures and to understand any seasonal changes in patient characteristics. This exploration is timely in the context of increasing concern around high demand in ED throughout the year. The study is large, with over 1.5 million ED attendances and over 750,000 acute admissions, and demonstrates a considerable degree of robustness in that the same findings were derived across multiple variables, hospitals and research teams. The federated approach allowed use of existing local relationships and pipelines with data providers, rather than requiring a lengthy ‘from-scratch’ central data access request, and reduced risk by minimising the amount of record-level data viewable by any individual researcher.

The present study has several limitations, however. As noted above, we have not examined aspects of flow, only demand, and we have not examined whether ambulance arrivals or handover times may more accurately reflect seasonal pressures. We only examined the primary diagnosis in each ED and admission record to examine seasonal diagnosis. This choice was taken to facilitate as concise an analysis as possible, and to focus on the main reason for attendance or admission, but we may have seen a greater difference if we had examined all diagnoses. Additionally, we did not directly examine some other diagnoses that may vary seasonally, such as falls, which could concievably affect patient flow. We also excluded unplanned follow-up ED attendances within 7 days of the initial unplanned attendance. Such attendances could potentially show some seasonality, though these account for only around 2% of attendances.

Finally, the nature of the federated analysis made in-depth, multivariable modelling approaches unfeasible for this study, meaning that most of our findings are based on summary statistics and over only one year. Complex relationships between seasonality and outcomes would not be revealed, and results may not represent recent years overall.

### Future research

Many of the limitations above may be addressed with additional studies. Studies utilising prospective data collection and qualitative components to contextualise findings would be able to address concerns about existing mitigations obscuring seasonal pressure, and to explore a range of outcomes not currently collected within routine datasets. A replication of this study using the CUREd+ database [36] is currently planned. This database contains linked, record-level information regarding ED attendances and acute admission for all of England from 2014 to 2024, and also includes ambulance data for the Yorkshire and Humber region. This will allow analysis of more sites across the country, additional outcomes and contact types (including unplanned follow-up ED attendances and ambulance arrivals), and over multiple years. In turn this will help to reveal differences across years and to explore key variables in more detail than was possible using this federated approach.

## Conclusions

This study suggests that pressures in ED are significant all year round to the extent that what were considered ‘winter pressures’ are not revealed by routinely collected data and may no longer be seasonal. Future research will aim to replicate and contextualise these findings in a wider sample using more in-depth analysis. Policymakers should consider extending seasonal mitigation measures and implementing evidence-based structural adjustments - such as increasing primary care provision - to help reduce and manage demand all year.

## Supplementary Information

Below is the link to the electronic supplementary material.

Supplementary Material 1: SAP & Template tables. PDF file containing the Statistical analysis plan and example extracts of the template summary tables supplied to regional analysis teams.

Supplementary Material 2: Variable encoding and categorisation. PDF file containing tables detailing the categorisation of variables and code mappings for the analysis.

Supplementary Material 3: Chief complaint coding & categorisation. PDF file containing list of SNOMED chief complaint codes and the categories used for the analysis.

Supplementary Material 4: Ambulatory Emergency Care Conditions. PDF File containing list of conditions considered Ambulatory Emergency Care Conditions and their associated ICD10 code.

Supplementary Material 5: Additional results. PDF File containing figures and tables documenting further results not presented in the main text.

## Data Availability

Data for this project is owned and administered by multiple NHS trusts and is not publicly available. To facilitate knowledge in this area, the anonymised participant data for Birmingham, analytical code and a data dictionary defining each field will be available to others through application to PIONEER Data Hub via the corresponding author. Source data from Southampton can be made available to the hospital upon request. Requests for data for all other regions should be made to the appropriate NHS trusts.

## Abbreviations

AECC: Ambulatory emergency care condition
APC: Admitted patient care
ECDS: Emergency care data set
ED: Emergency department
LOS: Length of stay
NHS: National health service
NHS 111: National health service 111 telephone triage service
UEC: Urgent and emergency care
