# Supplementary material for: Winter all year round in urgent and emergency care: a large retrospective analysis of routinely collected NHS data across England, 2021–2022
Source: BMC Health Serv Res. 2026 Mar 4;26:499. doi: 10.1186/s12913-026-14253-3 (PMC13067658; doi:10.1186/s12913-026-14253-3)
Supplement: Supplementary file 1 — Supplementary Material 1: SAP & Template tables. PDF file containing the Statistical analysis plan and example extracts of the template summary tables supplied to regional analysis teams. [file 12913_2026_14253_MOESM1_ESM.pdf]

Additional File 1: SAP & Template tables

SUMMARY:
We have seen a large increase in waiting times for emergency care across the country. It is not very clear why this is happening, and in order to understand how we can solve the problem, we need to understand this demand and where it is coming from.

This study will look at two key areas that are experiencing strain at the moment. Firstly, it will examine anonymised routine data from NHS Emergency Departments (ED) across the country. We will look at patterns of patients attending the ED, who they are, what medical complaints they arrive with and what happens to them. We will also use a tried and tested approach to identifying which patients come to the ED, that could reasonably have been seen by a GP or other non-emergency health service. Secondly the study will examine emergency admissions to hospital to understand who is being admitted to hospital and what happens to them. We will also use established codes to identify patients being admitted with conditions that could be diagnosed and treated in the same day (called Ambulatory Emergency Care Conditions).

We plan to see what the variation is in ED attendance and hospital admission between different regions, and at different times of the year to understand greater pressures on emergency care in the winter. We will use our findings to help identify groups of patients where a different pathway of care might improve their experiences and outcomes. This will help us to plan how health services need to be redesigned to meet the needs of the population in a more efficient and effective way.

AIMS:

1. To understand the current demand on emergency departments and hospital admissions and how does this vary nationally and throughout the year
2. To explore the demographic factors driving demand for emergency care, particularly in the Winter months
3. To explore which attendances to the emergency department are avoidable, and whether their number and characteristics vary throughout the year
4. To explore which acute admissions to hospital are avoidable, and do their number and characteristics vary throughout the year

Objectives

1. Use regional datasets of routine hospital data to define the type of emergency demand, what the national picture is and how this varies by region and season
2. Take an algorithmic approach to describing adults attending the emergency department avoidably
3. Use Ambulatory Emergency Care Condition codes to define adults being admitted to hospital avoidably
4. Establish a clearer picture of how demand is being created across regions and over the year that can indicate where solutions may lie and propose next steps

This study will access and analyse existing routine NHS datasets. These include 12 months of data from 10 regions (sites) including 20 Type I EDs and associated acute hospitals. The study is in two parts using the data to provide a more holistic picture of both demand through ED and acute admission pathways.

1. ECDS (ED) dataset:

1.1 Analysis strata

This section describes the analysis strata for the analysis of the ECDS data. These will be referred to in the analysis descriptions that follow.

1.1.1 Attendances by season

This analysis population includes all unplanned first emergency care attendances for a new clinical condition (or deterioration of a chronic condition) at Type 1 Emergency Departments (Emergency Departments that are a consultant led 24 hour service with full resuscitation facilities and designated accommodation for the reception of emergency care patients)

Filter: (edattendcat = 1) & (eddepttype = 01)

These filters should have been applied to the original data extract.

Analysis will stratify by season according to two exclusive definitions:
i) Six-month winter definition

- Winter: 1st October-31st March inclusive (Note start date of data period is 1st Nov 2021; end date is 31st Oct 2022)
    - (**edarrivaldatetime** > “2021/10/31 23:59:59” & **edarrivaldatetime** < “2022/04/01 00:00:00”) | (**edarrivaldatetime** > 2022/09/30 23:59:59 & **edarrivaldatetime** > 2022/11/01 00:00:00)
- Non-winter: 1st April-30th September inclusive
    - (**edarrivaldatetime** > “2022/03/31 23:59:59” & **edarrivaldatetime** < “2022/10/01 00:00:00”)

ii) Three-month winter definition

- Winter: 1st December-28th February inclusive (given 2022 not a leap year)
    - (**edarrivaldatetime** > “2021/11/30 23:59:59” & **edarrivaldatetime** < “2022/03/01 00:00:00”)
- Non-Winter: 1st March-30th November inclusive
    - (**edarrivaldatetime** > “2021/10/31 23:59:59” & **edarrivaldatetime** < “2022/12/01 00:00:00”) | (**edarrivaldatetime** > “2022/02/28 23:59:59” & **edarrivaldatetime** < “2022/11/01 00:00:00”)

1.1.2 Stratification by arrival mode
This analysis population further stratifies that described in section 1.1.1. In addition to exploration by season, this will stratify according to arrival mode:

- Arrival by ambulance
    - **edarrivalmode** == Ambulance
- Arrival by other means
    - **edarrivalmode** != Ambulance

1.1.3 Stratification by time of arrival
This analysis population further stratifies that described in section 1.1.1. In addition to exploration by season, this will stratify according to time of arrival:

- In-hours arrival: (8am-5:59pm, Monday-Friday)
    - (**Day of Week** in Monday, Tuesday, Wednesday, Thursday, Friday) & (**Time of Day** > 07:59:59 & **Time of Day** < 18:00:00)
- Out of hours arrival: (Saturday, Sunday, or 6pm-7:59am Monday-Friday)
    - (**Day of Week** in Saturday, Sunday) | ((**Day of Week** in Monday, Tuesday, Wednesday, Thursday, Friday) & (**Time of Day** < 08:00:00 | **Time of Day** > 17:59:59))

1.1.4 Stratification by attendance urgency
This analysis population further stratifies that described in section 1.1.1. In addition to exploration by season, this will stratify according to the calculated urgency of the attendance

- Urgent attendance
- Non-urgent attendance

1.2 Summary Statistics
Summary statistics of the patient demographics, attendance characteristics and attendance outcomes will be calculated. For numerical variables, the minimum (min), maximum (max), mean, standard deviation (SD), median,

lower quartile (Q1) and upper quartile (Q3) will be presented with the number of observations used in the calculations. For categorical variables, the number and percentage of patients in each of the categories and the total number of observations will be calculated.

Summary statistics for the demographic variables will be presented at the hospital site level (identified using **edsitecode**). This analysis will allow the comparison of demographics between different Emergency Departments and investigate any variation in discharge destination (e.g. variation in admissions).

The following summaries will be presented for analysis populations:

- **All attendances October-March**
- **All attendances April-September**
- **All attendances December-February**
- **All attendances March-November**
    - **All seasonal analysis additionally stratified by arrival mode**
    - **All seasonal analysis additionally stratified by time of arrival**
    - **All seasonal analysis additionally stratified by urgency of attendance**

1.2.1 Patient Demographics

- Age (categorical (to be decided)) - Frequency and proportions
- Age (continuous) - (N, min, max, mean, SD, median, Q1, Q3)
- Gender - Frequency and proportions
- Ethnicity (Categories to be decided) - Frequency and proportions
- Townsend score decile - Frequencies and proportions
- Care home flag - Frequencies and proportions
- Comorbidities - Frequencies and proportions

1.2.2 Attendance Characteristics

- Arrival mode - Frequencies and proportions (Not to be calculated for arrival mode stratification analysis)
- Source of attendance (Categories to be decided) - Frequencies and proportions
- Time of arrival (In hours; out-of-hours) - Frequencies and proportions (Not to be calculated for time of arrival analysis)
- Acuity - Frequencies and proportions
- Presenting complaint - Frequencies and proportions
- Seasonal diagnosis - Frequencies and proportions
- Investigations - Frequencies and proportions
- Treatments - Frequencies and proportions
- Certainty - Frequencies and proportions
- Discharge destination - Frequencies and proportions

1.2.3 Attendance Outcomes

- Time in department - (N, min, max, mean, SD, median, Q1, Q3)
- ED waiting time - (N, min, max, mean, SD, median, Q1, Q3)
- Non-urgent investigations per attendance - (N, min, max, mean, SD, median, Q1, Q3)
- Urgent investigations per attendance - (N, min, max, mean, SD, median, Q1, Q3)
- Non-urgent treatments per attendance - (N, min, max, mean, SD, median, Q1, Q3)
- Urgent treatments per attendance - (N, min, max, mean, SD, median, Q1, Q3)

1.3 Regression analysis

Regression analyses will examine the impact of season on the following outcomes

- ED waiting time (minutes; continuous)
- Total time in ED (minutes; continuous)
- Number of investigations performed (Binary: <=1; >1)
- Number of treatments performed (Binary: <=1; >1)

ED waiting time and Total time in ED will be modelled using linear regression. Number of investigations and treatments will be modelled using logistic regression.

Each model will take season as a main predictor, using six-month and three-month definitions of winter in separate analyses.

Each regression will include the following covariates:

- Age (continuous)
- Gender (Reference category = Male. Collapse indeterminate/unknown into single category)
- Ethnicity (Reference category = White. Collapse other/not stated/not known into single category)
- Deprivation quintile (Reference category = 1)
- Care home flag (Binary. Reference category = No)
- Seasonal Diagnosis group (Reference category = None)
- Time (binary in/out of hours. Reference category = in-hours)

2. Admitted Patient Care (APC) Dataset

2.1 Analysis strata
This section describes the analysis strata for the analysis of the APC data. These populations will be referred to in the analysis descriptions that follow.

2.1.1 Acute admissions by season
This analysis population includes all acute admissions.

Filter: (admimeth in 21, 22, 23, 24, 25, 2A, 2B, 2C, 2D, 28) & (epiorder = 1)
These filters should have been applied to the original data extract.

Analysis will stratify by season according to two exclusive definitions:
i) Six-month winter definition

- Winter: 1st October-31st March inclusive (Note start date of data period is 1st Nov 2021; end date is 31st Oct 2022)
    - (**admidate** > “2021/10/31” & **admidate** < “2022/04/01”) | (**admidate** > 2022/09/30 & **admidate** < 2022/11/01)
- Non-winter: 1st April-30th September inclusive
    - (**admidate** > “2022/03/31” & **admidate** < “2022/10/01”)

ii) Three-month winter definition

- Winter: 1st December-28th February inclusive (given 2022 not a leap year)
    - (**admidate** > “2021/11/30” & **admidate** < “2022/03/01”)
- Non-Winter: 1st March-30th November inclusive
    - (**admidate** > “2021/10/31” & **admidate** < “2022/12/01”) | (**admidate** > “2022/02/28” & **admidate** < “2022/11/01”)

2.1.1 Stratification by discharge destination
This analysis population further stratifies that described in section 2.1.1. In addition to exploration by season, this will stratify according to discharge destination:

- Discharge to care home
    - **disdest** == “Care Home”
- Discharge to medical care
    - **disdest** == “Medical Care”
- Discharge other
    - **disdest** != “Care Home” & **disdest** != “Medical Care”

2.1.2 Stratification by time of admission
This analysis population further stratifies that described in section 2.1.1. In addition to exploration by season, this will stratify according to time of admission:

- In-hours arrival: (8am-5:59pm, Monday-Friday)

    - (**Day of Week** in Monday, Tuesday, Wednesday, Thursday, Friday) & (**Time of Day** > 07:59:59 & **Time of Day** < 18:00:00)
- Out of hours arrival: (Saturday, Sunday, or 6pm-7:59am Monday-Friday)
    - (**Day of Week** in Saturday, Sunday) | ((**Day of Week** in Monday, Tuesday, Wednesday, Thursday, Friday) & (**Time of Day** < 08:00:00 | **Time of Day** > 17:59:59))

2.1.3 Stratification by Avoidability (AECC)
This analysis population further stratifies that described in section 2.1.1. In addition to exploration by season, this will stratify according to the avoidability of the admission, defined as an Ambulatory Emergency Care Condition (AECC).

- Potentially avoidable admission (AECC)
- Non-avoidable admission (Non-AECC)

AECC definitions can be found in the accompanying Avoidable Admissions project AECC code list.

2.2 Demographics
Summary statistics of the patient demographics, admission characteristics and admission outcomes will be calculated. For numerical variables, the minimum (min), maximum (max), mean, standard deviation (SD), median, lower quartile (Q1) and upper quartile (Q3) will be presented with the number of observations used in the calculations. For categorical variables, the number and percentage of patients in each of the categories and the total number of observations will be calculated.

Summary statistics for the demographic variables will be presented at the hospital site level (identified using **sitret**). This analysis will allow the comparison of demographics between different Hospitals.

The following summaries will be presented for analysis populations:

- **All admissions October-March**
- **All admissions April-September**
- **All admissions December-February**
- **All admissions March-November**
    - **All seasonal analysis additionally stratified by discharge destination**
    - **All seasonal analysis additionally stratified by time of admission**
    - **All seasonal analysis additionally stratified by avoidability (AECC)**

2.2.1 Patient Demographics

- Age (categorical (to be decided)) - Frequency and proportions
- Age (continuous) - (N, min, max, mean, SD, median, Q1, Q3)
- Gender - Frequency and proportions
- Ethnicity (Categories to be decided) - Frequency and proportions
- Townsend score decile - Frequencies and proportions
- Care Home Flag - Frequencies and proportions
- Comorbidities - Frequencies and proportions

2.2.2 Admission Characteristics

- Source of admission - Frequencies and proportions
- Time of admission - (In hours; out of hours) - Frequencies and proportions (Not to be calculated for time of admission analysis)
- Seasonal diagnosis - Frequencies and proportions
- Discharge destination - Frequencies and proportions

2.2.3 Admission Outcomes

- Length of stay - (N, min, max, mean, SD, median, Q1, Q3)
- Length of stay dichotomised (<2 days)

- Procedures per attendance - (N, min, max, mean, SD, median, Q1, Q3)
- Procedures dichotomised (<2 procedures)
- Days ready for discharge - (N, min, max, mean, SD, median, Q1, Q3)
- Days ready for discharge dichotomised (<2 days)
- Admission type - Frequencies and proportions

2.3 Regression analysis

Regression analyses will examine the impact of season on the following outcomes

- Length of stay (Binary; <2 days; >= 2 days)
- Number of procedures performed (Binary: <2; >=2)
- Days ready for discharge (Binary: <2; >=2)
- Avoidable admission (Binary; Yes; No)

All outcomes will be modelled using logistic regression.

Each model will take season as a main predictor, using six-month and three-month definitions of winter in separate analyses.

Each regression will include the following covariates:

- Age (continuous)
- Gender (Reference category = Male. Collapse indeterminate/unknown into single category)
- Ethnicity (Reference category = White. Collapse other/not stated/not known into single category)
- Deprivation quintile (Reference category = 1)
- Care home flag (Binary. Reference category = No)
- Seasonal Diagnosis group (Reference category = None)
- Time (binary in/out of hours. Reference category = in-hours)

Template analysis tables (example extract only)

ED Analysis
As indicated in tables, produce

I. For each seasonal strata overall;
II. Additionally stratified by arrival mode (ambulance/non-ambulance)
III. Additionally stratified by time of arrival (out-of-hours/in-hours)
IV. Additional stratified by urgency (urgent/non-urgent)

Create all following tables for each hospital site (identified using **edsitecode**).
All outputs for Type 1 EDs only (**eddepttye** == 01) and first unplanned ED attendance for new condition only (**edattendcat** == 01)

Patient Demographics
6-month Winter

	**Attendances Oct-March**								**Attendances Apr-Sept**							
	N	min	max	mean	SD	median	Q1	Q3	N	min	max	mean	SD	median	Q1	Q3
**Age**																

	6-month Winter			
	**Attendances Oct-March**		**Attendances Apr-Sept**	
	N	(%)	N	(%)
< 20 years<br>20 - 24<br>25 - 29<br>30 - 34<br>35 - 39<br>40 - 44				

45 - 49<br>50 - 54<br>55 - 59<br>60 - 64<br>65 - 69<br>70 - 74<br>75 - 79<br>80 - 84<br>85+<br>Not Known				
Male<br>Female<br>Indeterminate<br>Unknown				
White<br>Mixed<br>Asian or Asian British<br>Black or Black British<br>Other Ethnic Groups<br>Not stated<br>Not known				
Quintile 1 (least deprived)<br>Quintile 2<br>Quintile 3<br>Quintile 4<br>Quintile 5 (most deprived)				
Yes<br>No<br>Unknown				
Yes<br>No				

Attendance Characteristics

	6-month Winter	
	**Attendances Oct-March**	**Attendances Apr-Sept**

	N	(%)	N	(%)
Walk-In<br>Ambulance<br>Other				
Personal<br>Primary Care<br>Hospital<br>Emergency Services				
In-hours				
Out-of-hours				
Immediate care<br>Very urgent<br>Urgent<br>Standard<br>Low acuity				
Airway / breathing<br>Circulation / chest<br>…<br>…<br>General / minor / admin				
Respiratory infection<br>Respiratory exacerbation<br>None				
None<br>Non-urgent<br>Urgent				
None<br>Non-urgent<br>Urgent				
Suspected diagnosis<br>Confirmed diagnosis				
Discharged<br>Ambulatory / Short Stay				

Admitted<br>Transfer<br>Died				

Attendance Outcomes

6-month Winter

	Attendances Oct-March								Attendances Apr-Sept							
	N	min	max	mean	SD	median	Q1	Q3	N	min	max	mean	SD	median	Q1	Q3
Time in department																
ED waiting time																
Non-urgent investigations (per attendance)																
Urgent investigations (per attendance)																
Non-urgent treatments (per attendance)																
Urgent treatments (per attendance)																

	6-month Winter			
	**Attendances Oct-March**		**Attendances Apr-Sept**	
	N	(%)	N	(%)
Urgent				

Non-urgent				
