# Supplementary material for: Winter all year round in urgent and emergency care: a large retrospective analysis of routinely collected NHS data across England, 2021–2022
Source: BMC Health Serv Res. 2026 Mar 4;26:499. doi: 10.1186/s12913-026-14253-3 (PMC13067658; doi:10.1186/s12913-026-14253-3)
Supplement: Supplementary file 2 — Supplementary Material 2: Variable encoding and categorisation. PDF file containing tables detailing the categorisation of variables and code mappings for the analysis. [file 12913_2026_14253_MOESM2_ESM.pdf]

Additional File 2: Variable encoding & categorisation

Details of encoding & categorisation of routine data fields provided to contributing research sites to support data extraction and analysis.

A2-1. ECDS Data

Age

Data spec variable: **activage** (AGE AT CDS ACTIVITY DATE (data dictionary.nhs.uk))

Code	Description
999	Not known i.e. date of birth not known and age cannot be estimated
1	< 20 years
2	20 - 24
3	25 - 29
4	30 - 34
5	35 - 39
6	40 - 44
7	45 - 49
8	50 - 54
9	55 - 59
10	60 - 64
11	65 - 69
12	70 - 74
13	75 - 79
14	80 - 84

15	85+
999	Not Known

Gender

Data spec variable: **gender** (PERSON STATED GENDER CODE (data dictionary.nhs.uk))

Code	Description
1	Male
2	Female
9	Indeterminate (unable to be classified as either male or female)
X	Not Known

Ethnicity

Data spec variable: **ethnos** (ETHNIC CATEGORY (datadictionary.nhs.uk))

Code	Description	Categories
A	White - British	White
B	White - Irish	White
C	White - Any other White background	White
D	Mixed - White and Black Caribbean	Mixed
E	Mixed - White and Black African	Mixed
F	Mixed - White and Asian	Mixed
G	Mixed - Any other mixed background	Mixed
H	Asian or Asian British - Indian	Asian or Asian British
J	Asian or Asian British - Pakistani	Asian or Asian British

K	Asian or Asian British - Bangladeshi	Asian or Asian British
L	Asian or Asian British - Any other Asian background	Asian or Asian British
M	Black or Black British - Caribbean	Black or Black British
N	Black or Black British - African	Black or Black British
P	Black or Black British - Any other Black background	Black or Black British
R	Other Ethnic Groups - Chinese	Other Ethnic Groups
S	Other Ethnic Groups - Any other Ethnic Groups	Other Ethnic Groups
Z	Not stated (e.g. unwilling to state)	Not stated
99	Not known (e.g. unconscious)	Not known

Townsend Score Decile

Data spec variable: **townsend_score_quintile (**2011 UK Townsend Deprivation Scores - Dataset - UK Data Service CKAN**)**

Code	Description
1	Quintile 1 (least deprived)
2	Quintile 2
3	Quintile 3
4	Quintile 4
5	Quintile 5 (most deprived)

Care Home Flag

Data spec variable: **AccommodationStatus_SnomedCt** (ACCOMMODATION STATUS (SNOMED CT))
This variable will indicate where a patient was resident in a care home prior to arrival at ED.

SNOMED CODE	ECDS Description with notes	Care Home
414418009	**Patient has own stable accommodation e.g. home / flat**<br>Includes : house, farm house, non-institutional place of residence, apartment/ flat, boarding house, hotel, caravan park, refuge, long-term squat with utilities<br>Excludes : Institutional long-term place of residence, Abandoned or derelict house	No
224221006	**Warden controlled accommodation**<br>Includes : home with intermittent welfare checks<br>Excludes : residential / nursing home with continuous dedicated staffing	No
394923006	**Residential institution WITHOUT routine nursing care**<br>Includes : Children's home, residential home, old people's home, military camp, prison, monastery<br>Excludes : Hospital, nursing home, hospice, psychiatric hospital	No
160734000	**Residential institution WITH routine nursing care**<br>Includes : nursing home, hospice<br>Excludes : hospital, residential home, psychiatric hospital	Yes
224225002	**Medical area**<br>Includes : hospital, clinic, psychiatric hospital (long term)<br>Excludes : hospice, nursing home	No
224231004	**Homeless in night shelter**<br>Includes : night shelter, homeless shelter, emergency housing<br>Excludes : sleeping rough	No
32911000	**Homeless without accommodation**<br>Includes : homeless, sleeping rough, abandoned or derelict housing, squatting without utilities<br>Excludes : night shelter, homeless shelter	No
1064831000000106	**Usual accommodation not given: patient refused**<br>Includes : any situation where the patient can physically answer questions but refuses to answer this question.	Unknown
1064841000000102	**Usual accommodation not given: patient physically unable**<br>Includes : only situation when patient physically unable to respond e.g. unconscious and not able to establish by other means	Unknown
1066881000000100	**Usual accommodation not known**<br>Do not use unless there is no other applicable code	Unknown

Comorbidities

Data spec variable: edcomorb_NN (https://www.datadictionary.nhs.uk/data_elements/comorbidity__snomed_ct_.html)

Code	Category
Blank	No
Any SNOMED code	Yes

Arrival Mode

Data spec variable: **edarrivalmode** (EMERGENCY CARE ARRIVAL MODE (SNOMED CT))

SNOMED CODE	ECDS Description	Arrival Mode
1048071000000103	Patient arranged own transport / walk-in	Walk-In
1048061000000105	Arrival by public transport	Walk-In
1048031000000100	Emergency road ambulance	Ambulance
1048041000000109	Emergency road ambulance with medical escort	Ambulance
1048021000000102	Non-emergency road ambulance	Ambulance
1048051000000107	Helicopter	Ambulance
1048081000000101	Fixed wing / medical repatriation by air	Other
1047991000000102	Custodial services: prison / detention centre transport	Other
1048001000000106	Police Transport	Other

Source of Attendance

Data spec variable: **edattendsource** (EMERGENCY CARE ATTENDANCE SOURCE (SNOMED CT))

SNOMED CODE	ECDS Description	Categories
507291000000100	Self-referral to accident and emergency department (self/family/friends/education/work colleague)	Personal

1991000124105	Referred by self (self/family/friends/education/work colleague)	Personal
1065391000000104	Referred by carer (external, not family/friend)	Personal
315261000000101	Advised to attend accident and emergency department (non-NHS telephone/internet advice)	Perso.nal
276491000	Referred by member of Primary Health Care Team (GP/practice nurse)	Primary Care
108161000000109	Referred by general practitioner	Primary Care
166941000000106	Out of hours GP service	Primary Care
1082331000000106	Out of hours GP service	Primary Care
879591000000102	NHS telephone / internet advice e.g. NHS 111	Primary Care
1066431000000102	Emergency department	Hospital
1066441000000106	Urgent care service	Hospital
835091000000109	Outpatient service inc. ambulatory care	Hospital
835101000000101	Inpatient	Hospital
1465211000000108	Extended Care Episode	Hospital
1079521000000104	Private specialist	Hospital
185360007	Referred by hospital doctor	Hospital
1077191000000103	Community nurse (not practice nurse)	Community
1052681000000105	Health visitor	Community
185363009	Midwife	Community
1065401000000101	School nurse	Community
198251000000102	Referred by school	Community
1077201000000101	Community mental health nurse	Community
1065991000000100	Mental health assessment team	Community

877171000000103	Social services	Community
1077761000000105	Older persons day care centre	Community
1077211000000104	Homeless persons drop in centre	Community
1066011000000104	Custodial services : prison	Community
1066001000000101	Custodial services : detention centre	Community
185369008	Pharmacist (including community pharmacist)	Community
185366001	Dentist (including community dentist)	Community
185368000	Optician / optometrist	Community
1066021000000105	Advanced care practitioner	Emergency Services
198261000000104	Ambulance service - patient in transit	Emergency Services
889801000000100	Police service / forensic medical officer	Emergency Services
1066031000000107	Fire service	Emergency Services
1066061000000102	Search and rescue	Emergency Services
1066041000000103	Coastguard	Emergency Services
1066051000000100	Mountain rescue	Emergency Services

Acuity

Data spec variable: **edacuity** (EMERGENCY CARE ACUITY (SNOMED CT))

SNOMED Code	ECDS Description
1064891000000107	1 - Immediate care level emergency care
1064911000000105	2 - Very urgent level emergency care
1064901000000108	3 - Urgent level emergency care
1077241000000103	4 - Standard level emergency care

1077251000000100	5 - Low acuity level emergency care

Investigations

Data spec variables: **edinvest_01** to **edinvest_NN** (up to 12) EMERGENCY CARE CLINICAL INVESTIGATION (SNOMED CT)
NOTE: If all invest fields empty, data is considered missing. If one or more fields complete, empty fields are considered no investigation.

SNOMED CODE	ECDS Description	Categories
1088291000000101	None	None
Blank	None	Missing **OR** None
27171005	Urinalysis	Non-urgent
167252002	Urine pregnancy test	Non-urgent
67900009	Pregnancy test	Non-urgent
53115007	Dental investigation	Non-urgent
All others		Urgent

Treatments

Data spec variables: **edtreat_01** to **edtreat_NN** (up to 12) NHS Data Model EMERGENCY CARE PROCEDURE (SNOMED CT)
NOTE: If all edtreat fields empty, data is considered missing. If one or more fields complete, empty fields are considered no investigation.

SNOMED CODE	ECDS Description	Categories
183964008	None	None
Blank	None	None **OR** missing
266712008	Prescriptions	Non-urgent
413334001	Guidance / advice - written	Non-urgent
81733005	Dental treatment	Non-urgent
All others		Urgent

Seasonal Diagnosis

Data spec variable: **eddiag_01EmergencyCareDiagnosis_SnomedCt (**NHS Data Model EMERGENCY CARE DIAGNOSIS (SNOMED CT)**)**
NOTE: Only use first diagnosis recorded (eddiag_01) to record seasonal diagnosis

SNOMED CODE	ECDS Description	Categories
6142004	Influenza	Respiratory infection
233604007	Pneumonia	Respiratory infection
278516003	Lobar Pneumonia	Respiratory infection
205237003	Pneumonitis	Respiratory infection
50417007	Lower Respiratory Tract Infection	Respiratory infection
36971009	Sinusitis	Respiratory infection
90176007	Tonsilitis	Respiratory infection
62994001	Tracheitis	Respiratory infection
80384002	Epiglottitis	Respiratory infection
54150009	Upper Respiratory Infection	Respiratory infection
13645005	COPD - Chronic obstructive pulmonary disease	Chronic disease exacerbation
195951007	Acute exacerbation of chronic obstructive airways disease	Chronic disease exacerbation
195967001	Asthma	Chronic disease exacerbation
12295008	Bronchiectasis	Chronic disease exacerbation
1325171000000109	Acute COVID-19 infection	Respiratory infection
1325181000000106	Ongoing symptomatic COVID-19	Respiratory infection
1325161000000102	Post-COVID-19 syndrome	Respiratory infection
All others		None

Discharge Destination

Data spec variable: **edattenddispatch** (EMERGENCY CARE DISCHARGE DESTINATION (SNOMED CT))

SNOMED CODE	ECDS Description	Categories
306689006	Usual place of residence / family members	Discharged
306691003	Residential care facility without 24 hour nursing care (e.g. residential home)	Discharged
306694006	Residential care facility with 24 hour nursing care (e.g. nursing home)	Discharged
306705005	Police	Discharged
50861005	Custodial services e.g. prison / detention centre	Discharged
1066331000000109	Short stay (less than 24hr) ward outside the ED but managed by ED	Ambulatory / Short Stay
1066341000000100	Ambulatory Emergency Care service	Ambulatory / Short Stay
1066351000000102	Hospital in the home service	Ambulatory / Short Stay
306706006	Ward – physical ward bed outside ED	Admitted
1066361000000104	High Dependency Unit (level 2)	Admitted
1066371000000106	Coronary Care Unit (level 2)	Admitted
1066381000000108	Special Care Baby Unit (level 2)	Admitted
1066391000000105	Intensive Care Unit (level 3)	Admitted
1066401000000108	Neonatal Intensive Care Unit (level 3)	Admitted
19712007	Transfer to another hospital / healthcare facility	Transfer
183919006	Discharge to Hospice	Transfer
305398007	Mortuary	Died

Discharge status

Data spec variable: **disstatus** (EMERGENCY CARE DISCHARGE STATUS (SNOMED CT))

SNOMED CODE	ECDS Description	Categories
1077021000000100	Streamed from emergency department to general practitioner following initial assessment	Non-urgent
182992009	Treatment completed	Non-urgent
1066321000000107	Left care setting before treatment completed	Non-urgent
1066301000000103	Left care setting before initial assessment	Non-urgent
1066311000000101	Left care setting after initial assessment	Non-urgent
1077031000000103	Streamed from emergency department to urgent care service following initial assessment	Urgent
1077781000000101	Streamed to emergency department following initial assessment	Urgent
1324201000000109	Streamed from emergency department to inpatient unit following initial assessment	Urgent
1077081000000104	Streamed from emergency department to ambulatory emergency care service following initial assessment	Urgent
1077091000000102	Streamed from emergency department to falls service following initial assessment	Urgent
1077101000000105	Streamed from emergency department to frailty service following initial assessment	Urgent
1077041000000107	Streamed from emergency department to mental health service following initial assessment	Urgent
1077051000000105	Streamed from emergency department to dental service following initial assessment	Urgent
1077061000000108	Streamed from emergency department to ophthalmology service following initial assessment	Urgent
1077071000000101	Streamed from emergency department to pharmacy service following initial assessment	Urgent
63238001	Dead on arrival at hospital	Died
75004002	Emergency room admission, died in emergency room	Died

A2-2. APC Data (where different to ECDS data variables)

Source of Admission

Data spec variable: **admisorc** (SOURCE OF ADMISSION CODE (HOSPITAL PROVIDER SPELL))

Code	Description	Category
19	The usual place of residence, unless listed below, for example, a private dwelling whether owner occupied or owned by Local Authority, housing association or other landlord. This includes wardened accommodation but not residential accommodation where health care is provided. It also includes patients with no fixed abode.	Residence
29	Temporary place of residence when usually resident elsewhere, for example, hotels and residential educational establishments	Residence
39	Penal establishment, court or police station / police custody suite	Penal
49	NHS other hospital provider: high security psychiatric accommodation in an NHS hospital provider (NHS trust or NHS Foundation Trust)	Medical care
51	NHS other hospital provider: ward for general patients or the younger physically disabled or A&E department	Medical care
52	NHS other hospital provider: ward for maternity patients or neonates	Medical care
53	NHS other hospital provider: ward for patients who are mentally ill or have learning disabilities	Medical care
54	NHS run care home	Care Home
65	Local authority residential accommodation i.e. where care is provided	Care Home
66	Local authority foster care, but not in residential accommodation i.e. where care is provided	Residence
79	Babies born in or on the way to hospital	Residence
85	Non-NHS (other than local authority) run care home	Care Home
86	Non-NHS (other than local authority) run nursing home	Care Home
87	Non-NHS run hospital	Medical care
88	non-NHS (other than local authority) run hospice	Care Home
98	Not applicable	Unknown
99	Not known	Unknown

Seasonal Diagnosis

Data spec variable: **diag_01** (NHS Data Model PRIMARY DIAGNOSIS (ICD))

ICD10 CODE	Description	Categories
J09.X	Influenza due to identified zoonotic or pandemic influenza virus	Respiratory infection
J10.X	Influenza due to identified seasonal influenza virus	Respiratory infection
J11.X	Influenza, virus not identified	Respiratory infection
J12.X	Viral pneumonia, not elsewhere classified	Respiratory infection
J13.X	Pneumonia due to Streptococcus pneumoniae	Respiratory infection
J14.X	Pneumonia due to Haemophilus influenzae	Respiratory infection
J15.X	Bacterial pneumonia, not elsewhere classified	Respiratory infection
J16.X	Pneumonia due to other infectious organisms, not elsewhere classified	Respiratory infection
J17.X	Pneumonia in diseases classified elsewhere	Respiratory infection
J18.X	Pneumonia, organism unspecified	Respiratory infection
J20.X	Lower respiratory tract infection	Respiratory infection
J21.X	Lower respiratory tract infection	Respiratory infection
J22.X	Lower respiratory tract infection	Respiratory infection
J00.X	Acute nasopharyngitis [common cold]	Respiratory infection
J01.X	Acute sinusitis	Respiratory infection
J02.X	Acute pharyngitis	Respiratory infection
J03.X	Acute tonsillitis	Respiratory infection
J04.X	Acute laryngitis and tracheitis	Respiratory infection
J05.X	Acute obstructive laryngitis [croup] and epiglottitis	Respiratory infection

J06.X	Acute upper respiratory infections of multiple and unspecified sites	Respiratory infection
J40.X	Bronchitis, not specified as acute or chronic	Chronic disease exacerbation
J41.X	Simple and mucopurulent chronic bronchitis	Chronic disease exacerbation
J42.X	Unspecified chronic bronchitis	Chronic disease exacerbation
J43.X	Emphysema	Chronic disease exacerbation
J44.X	Other chronic obstructive pulmonary disease	Chronic disease exacerbation
J45.X	Asthma	Chronic disease exacerbation
J46.X	Status asthmaticus	Chronic disease exacerbation
J47.X	Bronchiectasis	Chronic disease exacerbation
U07.1	COVID-19, virus identified	Respiratory infection
U07.2	COVID-19, virus not identified	Respiratory infection
U10.X	Multisystem inflammatory syndrome associated with COVID-19	Respiratory infection
All others		None

Discharge Destination

Data spec variable: **disdest** (DISCHARGE DESTINATION CODE (HOSPITAL PROVIDER SPELL))

Code	Description	Category
19	The usual place of residence, including no fixed abode	Residence
29	Temporary place of residence when usually resident elsewhere, for example, hotels and residential educational establishments	Residence
30	Repatriation from high security psychiatric hospital	Medical care
37	Penal establishment - court	Penal
38	Penal establishment - police station	Penal

39	Penal establishment - court and police station excluded	Penal
48	High security psychiatric hospital, Scotland	Medical care
49	NHS other hospital provider - high security psychiatric	Medical care
50	NHS other hospital provider - medium secure unit	Medical care
51	NHS other hospital provider - ward for general PATIENTS or the younger physically disabled	Medical care
52	NHS other hospital provider - ward for maternity PATIENTS or Neonates	Medical care
53	NHS other hospital provider - ward for PATIENTS who are mentally ill or have learning disabilities	Medical care
54	NHS run Care Home	Care Home
65	Local Authority residential accommodation i.e. where care is provided	Care Home
66	Local Authority foster care	Residence
79	Not applicable - PATIENT died or still birth	Died
84	Non-NHS run hospital - medium secure unit	Medical care
85	Non-NHS (other than Local Authority) run Care Home	Care Home
87	Non-NHS run hospital	Medical care
88	Non-NHS (other than Local Authority) run Hospice	Care Home
98	Not applicable - Hospital Provider Spell not finished at episode end (i.e. not discharged) or current episode unfinished	Unknown
99	Not known	Unknown

Procedures

Data spec variables: **opertn_01** and **opertn_NN** (NHS Data Model PRIMARY PROCEDURE (OPCS) and NHS Data Model PROCEDURE (OPCS) respectively)

**Code**	**Category**
- = No procedures performed	No

4an = Procedure code	Yes
& = Not known<br>X998 = Procedure carried out but no appropriate OPCS-4 code available (submitted value present between 1997-98 and 2005-07)<br>X999 = No procedure carried out (submitted value present between 1997-98 and 2001-02)	Missing
