# Supplementary material for: Winter all year round in urgent and emergency care: a large retrospective analysis of routinely collected NHS data across England, 2021–2022
Source: BMC Health Serv Res. 2026 Mar 4;26:499. doi: 10.1186/s12913-026-14253-3 (PMC13067658; doi:10.1186/s12913-026-14253-3)
Supplement: Supplementary file 3 — Supplementary Material 3: Chief complaint coding & categorisation. PDF file containing list of SNOMED chief complaint codes and the categories used for the analysis. [file 12913_2026_14253_MOESM3_ESM.pdf]

Additional File 3: Chief complaint coding & categorisation

SNOMED Code	SNOMED UK Preferred Term	Chief Complaint Category
13094009	Apnoea in the newborn	Airway / breathing
66857006	Haemoptysis	Airway / breathing
70407001	Stridor	Airway / breathing
87317003	Respiratory arrest	Airway / breathing
230145002	Difficulty breathing	Airway / breathing
248573009	Noisy respiration	Airway / breathing
262599003	Foreign body in respiratory tract	Airway / breathing
267036007	Dyspnoea	Airway / breathing
29857009	Chest pain	Circulation / chest
80313002	Palpitations	Circulation / chest
271594007	Syncope	Circulation / chest
361137007	Irregular heart beat	Circulation / chest
410429000	Cardiac arrest	Circulation / chest
422970001	Cardiac arrest due to trauma	Circulation / chest
427461000	Near syncope	Circulation / chest
449614009	Swelling of lower limb	Circulation / chest
762898005	Swelling of bilateral lower limbs	Circulation / chest
21631000119105	Limb ischaemia	Circulation / chest
271787007	Collapse	Circulation / chest
309585006	Syncope and collapse	Circulation / chest
386705008	Lightheadedness	Circulation / chest
419045004	Loss of consciousness	Circulation / chest
449615005	Swelling of lower leg	Circulation / chest
18653004	Alcohol intoxication delirium	Drug and Alcohol
66214007	Substance misuse	Drug and Alcohol
191480000	Alcohol withdrawal syndrome	Drug and Alcohol
363101005	Drug withdrawal	Drug and Alcohol
75478009	Poisoning	Environmental
87970004	Nonfatal submersion	Environmental
370977006	Frostbite	Environmental
371704001	Injury due to chemical exposure	Environmental
371708003	Injury due to electrical exposure	Environmental
386689009	Hypothermia	Environmental
417981005	Exposure to blood and/or body fluid	Environmental
418723004	Effects of direct lightning strike	Environmental
426936004	Smoke inhalation injury	Environmental
444107005	Exposure to communicable disease	Environmental
1156755000	Poisoning caused by gaseous substance	Environmental
10850741000119108	Accidental needle stick injury	Environmental
57335002	Toxic effect of gas, fumes AND/OR vapours	Environmental
41652007	Pain in eye	Eye
55899000	Foreign body on external eye	Eye
63102001	Visual disturbance	Eye
75705005	Red eye	Eye

170720001	Follow-up ophthalmological assessment	Eye
246679005	Discharge from eye	Eye
282752000	Injury of eye region	Eye
409668002	Photophobia	Eye
8765009	Haematemesis	Gastrointestinal
14760008	Constipation	Gastrointestinal
18165001	Jaundice	Gastrointestinal
21522001	Abdominal pain	Gastrointestinal
33334006	Foreign body in digestive tract	Gastrointestinal
40739000	Dysphagia	Gastrointestinal
47609003	Foreign body in oesophagus	Gastrointestinal
60728008	Swollen abdomen	Gastrointestinal
62315008	Diarrhoea	Gastrointestinal
65958008	Hiccoughs	Gastrointestinal
70176004	Foreign body in rectum	Gastrointestinal
77880009	Rectal pain	Gastrointestinal
79890006	Loss of appetite	Gastrointestinal
276464002	Injury of anus	Gastrointestinal
405729008	Blood in stool	Gastrointestinal
422400008	Vomiting	Gastrointestinal
422587007	Nausea	Gastrointestinal
3415004	Cyanosis	General
13791008	Asthenia	General
78680009	Hospital admission, emergency, direct	General
80394007	Hyperglycaemia	General
84387000	Asymptomatic	General
161152002	Social problem	General
162214009	Crying infant	General
182888003	Medication requested	General
302866003	Hypoglycaemia	General
385486001	Postoperative complication	General
386661006	Fever	General
410379003	Dressing change/wound care surveillance	General
473023007	Complication associated with device	General
162743000	Blue lips	General
226007004	Post-surgical wound care	General
288945004	Difficulty swallowing food	General
225358003	Wound care	General
6923002	Injury of perineum	Genitourinary
20502007	Pain in scrotum	Genitourinary
34436003	Blood in urine	Genitourinary
49650001	Dysuria	Genitourinary
56890008	Victim of sexual aggression	Genitourinary
162116003	Increased frequency of urination	Genitourinary
162143008	Pain in female genitalia	Genitourinary
225565007	Perineal pain	Genitourinary
247355005	Flank pain	Genitourinary
267064002	Retention of urine	Genitourinary
279020002	Pain in male genitalia	Genitourinary

281398003	Groin mass	Genitourinary
282772005	Genital injury	Genitourinary
300528000	Penis problem	Genitourinary
718403007	Decreased urine output	Genitourinary
28442001	Polyuria	Genitourinary
63901009	Pain in testicle	Genitourinary
83128009	Oliguria	Genitourinary
274743004	Swelling of inguinal region	Genitourinary
2999009	Injury of ear	Head and neck
14380007	Foreign body in mouth	Head and neck
15188001	Hearing loss	Head and neck
19491003	Injury of nose	Head and neck
25479004	Foreign body in pharynx	Head and neck
27355003	Toothache	Head and neck
49727002	Cough	Head and neck
60862001	Tinnitus	Head and neck
68235000	Nasal congestion	Head and neck
74699008	Foreign body in nose	Head and neck
75441006	Foreign body in ear	Head and neck
81680005	Neck pain	Head and neck
95668009	Pain in face	Head and neck
162356005	Earache symptoms	Head and neck
249366005	Bleeding from nose	Head and neck
267102003	Sore throat symptom	Head and neck
300132001	Ear discharge	Head and neck
421581006	Pharyngeal swelling	Head and neck
16001004	Otalgia	Head and neck
7523003	Injury of thigh	Injury
52011008	Injury of finger	Injury
65978000	Injury of chest wall	Injury
82271004	Injury of head	Injury
90460009	Injury of neck	Injury
125593007	Injury of face	Injury
125594001	Injury of shoulder region	Injury
125595000	Injury of upper arm	Injury
125596004	Injury of elbow	Injury
125597008	Injury of forearm	Injury
125598003	Injury of wrist	Injury
125599006	Injury of hand	Injury
125600009	Injury of hip region	Injury
125601008	Injury of knee	Injury
125602001	Injury of lower leg	Injury
125603006	Injury of ankle	Injury
125604000	Injury of foot	Injury
128069005	Injury of abdomen	Injury
262520005	Thumb injury	Injury
262525000	Chest injury	Injury
262595009	Traumatic amputation	Injury
282760004	Clavicle injury	Injury

282765009	Upper back injury	Injury
282766005	Lower back injury	Injury
282770002	Injury of ribs	Injury
282776008	Injury of toe	Injury
282780003	Heel injury	Injury
432754003	Injury of sternum	Injury
450724008	Injury of cervical region of back	Injury
1089261000000101	Major injury involving multiple body regions	Injury
2733002	Heel pain	Musculoskeletal
18876004	Pain in finger	Musculoskeletal
45326000	Shoulder pain	Musculoskeletal
47933007	Foot pain	Musculoskeletal
49218002	Hip pain	Musculoskeletal
53057004	Hand pain	Musculoskeletal
56608008	Pain in wrist	Musculoskeletal
74323005	Pain in elbow	Musculoskeletal
78514002	Thigh pain	Musculoskeletal
161891005	Backache	Musculoskeletal
247373008	Ankle pain	Musculoskeletal
271771009	Joint swelling	Musculoskeletal
285365001	Pain in toe	Musculoskeletal
300954003	Pain in calf	Musculoskeletal
300955002	Pain in thumb	Musculoskeletal
444899003	Pain in forearm	Musculoskeletal
771083005	Pain in upper arm	Musculoskeletal
1003722009	Pain of knee region	Musculoskeletal
22631008	Unsteady when walking	Neurological
25064002	Headache	Neurological
26079004	Tremor	Neurological
29164008	Disturbance in speech	Neurological
40917007	Clouded consciousness	Neurological
44077006	Numbness	Neurological
91175000	Seizure	Neurological
95666008	Weakness of face muscles	Neurological
161898004	Falls	Neurological
193462001	Insomnia	Neurological
271782001	Drowsy	Neurological
404640003	Dizziness	Neurological
713514005	Muscle weakness of limb	Neurological
3006004	Disturbance of consciousness	Neurological
23168003	Speech dysfunction	Neurological
91019004	Paraesthesia	Neurological
249990003	Unsteady when standing	Neurological
430576002	At risk for injury due to fall	Neurological
34124000	Foreign body in vagina	ObGyn
289610003	Vaginal problem	ObGyn
290085007	Breast problem	ObGyn
301822002	Abnormal vaginal bleeding	ObGyn
428566005	Gestation less than 20 weeks	ObGyn

429715006	Gestation greater than 20 weeks	ObGyn
289530006	Vaginal bleeding	ObGyn
2073000	Delusions	Psychosocial / Behaviour change
6471006	Suicidal thoughts	Psychosocial / Behaviour change
7011001	Hallucinations	Psychosocial / Behaviour change
24199005	Feeling agitated	Psychosocial / Behaviour change
48694002	Anxiety	Psychosocial / Behaviour change
248004009	Physical aggression	Psychosocial / Behaviour change
248020004	Bizarre behaviour	Psychosocial / Behaviour change
248062006	Self-injurious behaviour	Psychosocial / Behaviour change
366979004	Depressed mood	Psychosocial / Behaviour change
427797008	Unusual change in behaviour	Psychosocial / Behaviour change
93459000	Foreign body in subcutaneous tissue	Skin
125666000	Burn	Skin
161887000	Spontaneous bruising	Skin
247441003	Erythema	Skin
271807003	Eruption	Skin
283682007	Bite - wound	Skin
297982009	Skin problem	Skin
299972003	Sting of skin	Skin
312608009	Laceration - injury	Skin
312609001	Puncture wound - injury	Skin
399963005	Abrasion	Skin
418363000	Itching of skin	Skin
444905003	Mass of soft tissue	Skin
93458008	Foreign body in skin	Skin
276438008	Swelling / lump finding	Skin
10601006	Pain in lower limb	Musculoskeletal
102556003	Pain in upper limb	Musculoskeletal
127278005	Injury of upper extremity	Injury
127279002	Injury of lower extremity	Injury
417746004	Traumatic injury	Injury
