# Supplementary material for: Winter all year round in urgent and emergency care: a large retrospective analysis of routinely collected NHS data across England, 2021–2022
Source: BMC Health Serv Res. 2026 Mar 4;26:499. doi: 10.1186/s12913-026-14253-3 (PMC13067658; doi:10.1186/s12913-026-14253-3)
Supplement: Supplementary file 4 — Supplementary Material 4: Ambulatory Emergency Care Conditions. PDF File containing list of conditions considered Ambulatory Emergency Care Conditions and their associated ICD10 code. [file 12913_2026_14253_MOESM4_ESM.pdf]

Additional File 4: Ambulatory Emergency Care Conditions

AEC Clinical Conditions	ICD10 Code
Abnormal liver function	C22.0
Abnormal liver function	C22.1
Abnormal liver function	C22.2
Abnormal liver function	C23.X
Abnormal liver function	C22.3
Abnormal liver function	C22.4
Abnormal liver function	C22.7
Abnormal liver function	C22.9
Abnormal liver function	C24.0
Abnormal liver function	C24.1
Abnormal liver function	C24.8
Abnormal liver function	C24.9
Abnormal liver function	C25.0
Abnormal liver function	C25.1
Abnormal liver function	C25.2
Abnormal liver function	C25.3
Abnormal liver function	C25.4
Abnormal liver function	C25.7
Abnormal liver function	C25.8
Abnormal liver function	C25.9
Abnormal liver function	C78.7
Abnormal liver function	D13.5
Abnormal liver function	D37.6
Abnormal liver function	K70.0
Abnormal liver function	K70.1
Abnormal liver function	K70.2
Abnormal liver function	K70.3
Abnormal liver function	K70.4
Abnormal liver function	K70.9
Abnormal liver function	K72.0
Abnormal liver function	K72.1
Abnormal liver function	K72.9
Abnormal liver function	K73.0
Abnormal liver function	K73.1
Abnormal liver function	K73.2
Abnormal liver function	K73.8
Abnormal liver function	K73.9
Abnormal liver function	K74.0
Abnormal liver function	K74.1
Abnormal liver function	K74.2
Abnormal liver function	K74.3
Abnormal liver function	K74.4
Abnormal liver function	K74.5
Abnormal liver function	K74.6

Abnormal liver function	K75.2
Abnormal liver function	K75.3
Abnormal liver function	K75.4
Abnormal liver function	K75.8
Abnormal liver function	K75.9
Abnormal liver function	K76.0
Abnormal liver function	K76.1
Abnormal liver function	K76.6
Abnormal liver function	K76.8
Abnormal liver function	K76.9
Abnormal liver function	K80.0
Abnormal liver function	K80.1
Abnormal liver function	K80.2
Abnormal liver function	K80.3
Abnormal liver function	K80.4
Abnormal liver function	K80.5
Abnormal liver function	K80.8
Abnormal liver function	K81.0
Abnormal liver function	K81.1
Abnormal liver function	K81.8
Abnormal liver function	K81.9
Abnormal liver function	K82.1
Abnormal liver function	K82.2
Abnormal liver function	K82.3
Abnormal liver function	K82.4
Abnormal liver function	K82.8
Abnormal liver function	K82.9
Abnormal liver function	K83.1
Abnormal liver function	K83.4
Abnormal liver function	K83.8
Abnormal liver function	K83.9
Abnormal liver function	K86.0
Abnormal liver function	K86.2
Abnormal liver function	K86.3
Abnormal liver function	K86.8
Abnormal liver function	K86.9
Abnormal liver function	K87.0
Abnormal liver function	K91.5
Abnormal liver function	R16.0
Abnormal liver function	R16.1
Abnormal liver function	R16.2
Abnormal liver function	R17.X
Abnormal liver function	R94.5
Acute headache	G43.0
Acute headache	G43.1
Acute headache	G43.2
Acute headache	G43.3
Acute headache	G43.8
Acute headache	G43.9

Acute headache	G44.0
Acute headache	G44.1
Acute headache	G44.3
Acute headache	G44.4
Acute headache	G44.8
Acute headache	G96.1
Acute headache	R51.X
Acute headache	G44.2
Anaemia	D46.0
Anaemia	D46.1
Anaemia	D46.2
Anaemia	D46.4
Anaemia	D46.7
Anaemia	D46.9
Anaemia	D50.0
Anaemia	D50.1
Anaemia	D50.8
Anaemia	D50.9
Anaemia	D51.0
Anaemia	D51.1
Anaemia	D51.2
Anaemia	D51.3
Anaemia	D51.8
Anaemia	D51.9
Anaemia	D52.0
Anaemia	D52.1
Anaemia	D52.8
Anaemia	D52.9
Anaemia	D53.1
Anaemia	D57.1
Anaemia	D58.0
Anaemia	D58.1
Anaemia	D58.2
Anaemia	D58.8
Anaemia	D58.9
Anaemia	D59.0
Anaemia	D59.1
Anaemia	D59.2
Anaemia	D59.4
Anaemia	D59.8
Anaemia	D59.9
Anaemia	D64.8
Anaemia	D64.9
Ascites	R18.X
Asthma	J45.0
Asthma	J45.1
Asthma	J45.8
Asthma	J45.9
Cellulitis of limb	I89.1

Cellulitis of limb	L01.0
Cellulitis of limb	L03.0
Cellulitis of limb	L03.1
Cellulitis of limb	L03.2
Cellulitis of limb	L03.3
Cellulitis of limb	L03.8
Cellulitis of limb	L03.9
Cellulitis of limb	L08.0
Cellulitis of limb	L08.8
Cellulitis of limb	L08.9
COPD	J21.0
COPD	J21.1
COPD	J21.8
COPD	J21.9
COPD	J40.X
COPD	J41.0
COPD	J42.X
COPD	J43.1
COPD	J43.2
COPD	J43.8
COPD	J43.9
COPD	J44.0
COPD	J44.1
COPD	J44.8
COPD	J44.9
LTRI/Community acquired pneumonia	J10.0
LTRI/Community acquired pneumonia	J11.0
LTRI/Community acquired pneumonia	J12.0
LTRI/Community acquired pneumonia	J12.1
LTRI/Community acquired pneumonia	J12.2
LTRI/Community acquired pneumonia	J12.3
LTRI/Community acquired pneumonia	J12.8
LTRI/Community acquired pneumonia	J12.9
LTRI/Community acquired pneumonia	J13.X
LTRI/Community acquired pneumonia	J14.X
LTRI/Community acquired pneumonia	J15.3
LTRI/Community acquired pneumonia	J15.4
LTRI/Community acquired pneumonia	J15.5
LTRI/Community acquired pneumonia	J15.6
LTRI/Community acquired pneumonia	J15.7
LTRI/Community acquired pneumonia	J15.8
LTRI/Community acquired pneumonia	J15.9
LTRI/Community acquired pneumonia	J16.0
LTRI/Community acquired pneumonia	J16.8
LTRI/Community acquired pneumonia	J17.0
LTRI/Community acquired pneumonia	J17.1
LTRI/Community acquired pneumonia	J17.8
LTRI/Community acquired pneumonia	J18.0
LTRI/Community acquired pneumonia	J18.1

LTRI/Community acquired pneumonia	J18.8
LTRI/Community acquired pneumonia	J18.9
LTRI/Community acquired pneumonia	J20.0
LTRI/Community acquired pneumonia	J20.1
LTRI/Community acquired pneumonia	J20.2
LTRI/Community acquired pneumonia	J20.3
LTRI/Community acquired pneumonia	J20.4
LTRI/Community acquired pneumonia	J20.5
LTRI/Community acquired pneumonia	J20.6
LTRI/Community acquired pneumonia	J20.7
LTRI/Community acquired pneumonia	J20.8
LTRI/Community acquired pneumonia	J20.9
LTRI/Community acquired pneumonia	J22.X
Congestive cardiac failure	I11.0
Congestive cardiac failure	I13.0
Congestive cardiac failure	I13.2
Congestive cardiac failure	I50.0
Congestive cardiac failure	I50.1
Congestive cardiac failure	I50.9
Congestive cardiac failure	J81
DVT	I80.1
DVT	I80.2
DVT	I80.3
DVT	I80.4
DVT	I81.22
DVT	M79.6
DVT	M79.8
Diabetes/Hypoglycaemia	E16.0
Diabetes/Hypoglycaemia	E16.1
Diabetes/Hypoglycaemia	E16.2
Diabetes/Hypoglycaemia	E10.0
Diabetes/Hypoglycaemia	E10.1
Diabetes/Hypoglycaemia	E10.2
Diabetes/Hypoglycaemia	E10.3
Diabetes/Hypoglycaemia	E10.4
Diabetes/Hypoglycaemia	E10.5
Diabetes/Hypoglycaemia	E10.6
Diabetes/Hypoglycaemia	E10.7
Diabetes/Hypoglycaemia	E10.8
Diabetes/Hypoglycaemia	E10.9
Diabetes/Hypoglycaemia	E11.0
Diabetes/Hypoglycaemia	E11.1
Diabetes/Hypoglycaemia	E11.2
Diabetes/Hypoglycaemia	E11.3
Diabetes/Hypoglycaemia	E11.4
Diabetes/Hypoglycaemia	E11.5
Diabetes/Hypoglycaemia	E11.6
Diabetes/Hypoglycaemia	E11.7
Diabetes/Hypoglycaemia	E11.8

Diabetes/Hypoglycaemia	E11.9
Diabetes/Hypoglycaemia	E13.2
Diabetes/Hypoglycaemia	E13.3
Diabetes/Hypoglycaemia	E13.4
Diabetes/Hypoglycaemia	E13.5
Diabetes/Hypoglycaemia	E13.6
Diabetes/Hypoglycaemia	E13.7
Diabetes/Hypoglycaemia	E13.8
Diabetes/Hypoglycaemia	E13.9
Diabetes/Hypoglycaemia	E14.2
Diabetes/Hypoglycaemia	E14.3
Diabetes/Hypoglycaemia	E14.4
Diabetes/Hypoglycaemia	E14.5
Diabetes/Hypoglycaemia	E14.6
Diabetes/Hypoglycaemia	E14.7
Diabetes/Hypoglycaemia	E14.8
Diabetes/Hypoglycaemia	E14.9
Electrolyte inbalance	E22.2
Electrolyte inbalance	E51.2
Electrolyte inbalance	E83.4
Electrolyte inbalance	E83.5
Electrolyte inbalance	E86.X
Electrolyte inbalance	E87.0
Electrolyte inbalance	E87.1
Electrolyte inbalance	E87.5
Electrolyte inbalance	E87.6
Electrolyte inbalance	E87.7
Electrolyte inbalance	E87.8
End of life care	Z51.5
Falls including syncope and collapse	I95.1
Falls including syncope and collapse	R26.8
Falls including syncope and collapse	R29.6
Falls including syncope and collapse	R54.X
Falls including syncope and collapse	R55.X
Falls including syncope and collapse	T67.1
First seizure/seizure in a known epileptic	R56.8
First seizure/seizure in a known epileptic	G25.3
First seizure/seizure in a known epileptic	G40.0
First seizure/seizure in a known epileptic	G40.1
First seizure/seizure in a known epileptic	G40.2
First seizure/seizure in a known epileptic	G40.3
First seizure/seizure in a known epileptic	G40.4
First seizure/seizure in a known epileptic	G40.5
First seizure/seizure in a known epileptic	G40.6
First seizure/seizure in a known epileptic	G40.7
First seizure/seizure in a known epileptic	G40.8
First seizure/seizure in a known epileptic	G40.9
Gastroenteritis	A02.0
Gastroenteritis	A02.2

Gastroenteritis	A02.8
Gastroenteritis	A02.9
Gastroenteritis	A04.4
Gastroenteritis	A04.5
Gastroenteritis	A04.6
Gastroenteritis	A04.8
Gastroenteritis	A04.9
Gastroenteritis	A05.4
Gastroenteritis	A05.8
Gastroenteritis	A05.9
Gastroenteritis	A07.1
Gastroenteritis	A07.2
Gastroenteritis	A08.0
Gastroenteritis	A08.1
Gastroenteritis	A08.2
Gastroenteritis	A08.3
Gastroenteritis	A08.4
Gastroenteritis	A08.5
Gastroenteritis	A09.0
Gastroenteritis	A09.9
Gastroenteritis	K52.0
Gastroenteritis	K52.1
Gastroenteritis	K52.2
Gastroenteritis	K52.8
Gastroenteritis	K52.9
Gastroenteritis	A03.9
Inflammatory bowel disease	K50.0
Inflammatory bowel disease	K50.1
Inflammatory bowel disease	K50.8
Inflammatory bowel disease	K50.9
Inflammatory bowel disease	K51.0
Inflammatory bowel disease	K51.2
Inflammatory bowel disease	K51.3
Inflammatory bowel disease	K51.4
Inflammatory bowel disease	K51.5
Inflammatory bowel disease	K51.9
Inflammatory bowel disease	K52.3
Oesophageal stenosis	C15.0
Oesophageal stenosis	C15.1
Oesophageal stenosis	C15.2
Oesophageal stenosis	C15.3
Oesophageal stenosis	C15.4
Oesophageal stenosis	C15.5
Oesophageal stenosis	C15.8
Oesophageal stenosis	C15.9
Oesophageal stenosis	K22.0
Oesophageal stenosis	K22.2
Oesophageal stenosis	K22.4
Oesophageal stenosis	K22.5

Oesophageal stenosis	K22.7
Oesophageal stenosis	K22.8
Oesophageal stenosis	K22.9
Oesophageal stenosis	K23.8
Oesophageal stenosis	R12.X
Oesophageal stenosis	R13.X
Oesophageal stenosis	T18.1
Low risk acute kidney injury	N17.8
Low risk acute kidney injury	N17.9
Low risk acute kidney injury	N99.0
Low risk chest pain	I20.0
Low risk chest pain	I20.1
Low risk chest pain	I20.8
Low risk chest pain	I20.9
Low risk chest pain	I24.1
Low risk chest pain	I24.8
Low risk chest pain	I24.9
Low risk chest pain	I25.0
Low risk chest pain	I25.1
Low risk chest pain	I25.2
Low risk chest pain	I25.6
Low risk chest pain	I25.8
Low risk chest pain	I25.9
Low risk chest pain	M94.0
Low risk chest pain	M94.1
Low risk chest pain	R01.1
Low risk chest pain	R01.2
Low risk chest pain	R07.2
Low risk chest pain	R07.3
Low risk chest pain	R07.4
Low risk chest pain	Z03.4
Low risk chest pain	Z03.5
Other respiratory condiitons	E66.2
Other respiratory condiitons	J80.X
Other respiratory condiitons	J84.0
Other respiratory condiitons	J84.1
Other respiratory condiitons	J84.8
Other respiratory condiitons	J84.9
Other respiratory condiitons	J96.00
Other respiratory condiitons	J96.01
Other respiratory condiitons	J96.09
Other respiratory condiitons	J96.10
Other respiratory condiitons	J96.11
Other respiratory condiitons	J96.19
Other respiratory condiitons	J96.9
Other respiratory condiitons	J96.90
Other respiratory condiitons	J96.99
Other respiratory condiitons	J98.0
Other respiratory condiitons	J98.1

Other respiratory condiitons	J98.4
Other respiratory condiitons	J98.6
Other respiratory condiitons	J98.8
Other respiratory condiitons	J98.9
Other respiratory condiitons	J99.8
Other respiratory condiitons	Q34.0
Other respiratory condiitons	R04.2
Other respiratory condiitons	R04.9
Other respiratory condiitons	R05.X
Other respiratory condiitons	R06.0
Other respiratory condiitons	R06.2
Other respiratory condiitons	R06.4
Other respiratory condiitons	R09.8
Other respiratory condiitons	J47
Other respiratory condiitons	J64
Other respiratory condiitons	J67.9
PEG related complications	T85.5
PEG related complications	T85.8
PEG related complications	Z43.1
PEG related complications	K91.8
Pleural effusions	C78.2
Pleural effusions	J90.X
Pleural effusions	J91.X
Pleural effusions	J94.0
Pleural effusions	J94.8
Pneumothorax	J93.1
Pneumothorax	J93.8
Pneumothorax	J93.9
Pulmonary embolus	I26.0
Pulmonary embolus	I26.9
Pulmonary embolus	R07.1
Pulmonary embolus	R09.1
Self harm and accidental overdose	T36.X
Self harm and accidental overdose	T37.X
Self harm and accidental overdose	T38.X
Self harm and accidental overdose	T39.X
Self harm and accidental overdose	T40.X
Self harm and accidental overdose	T41.X
Self harm and accidental overdose	T42.X
Self harm and accidental overdose	T43.X
Self harm and accidental overdose	T44.X
Self harm and accidental overdose	T45.X
Self harm and accidental overdose	T46.X
Self harm and accidental overdose	T47.X
Self harm and accidental overdose	T48.X
Self harm and accidental overdose	T49.X
Self harm and accidental overdose	T50.X
Self harm and accidental overdose	T51.X
Self harm and accidental overdose	T52.X

Self harm and accidental overdose	T53.X
Self harm and accidental overdose	T54.X
Self harm and accidental overdose	T55.X
Self harm and accidental overdose	T56.X
Self harm and accidental overdose	T57.X
Self harm and accidental overdose	T58.X
Self harm and accidental overdose	T59.X
Self harm and accidental overdose	T60.X
Self harm and accidental overdose	T61.X
Self harm and accidental overdose	T62.X
Self harm and accidental overdose	T63.0
Self harm and accidental overdose	T63.1
Self harm and accidental overdose	T63.2
Self harm and accidental overdose	T63.3
Self harm and accidental overdose	T63.4
Self harm and accidental overdose	T64.X
Self harm and accidental overdose	T65.X
Supraventricular tachycardias	I44.0
Supraventricular tachycardias	I44.1
Supraventricular tachycardias	I44.4
Supraventricular tachycardias	I44.5
Supraventricular tachycardias	I44.6
Supraventricular tachycardias	I44.7
Supraventricular tachycardias	I45.0
Supraventricular tachycardias	I45.1
Supraventricular tachycardias	I45.2
Supraventricular tachycardias	I45.3
Supraventricular tachycardias	I45.4
Supraventricular tachycardias	I45.5
Supraventricular tachycardias	I45.6
Supraventricular tachycardias	I45.8
Supraventricular tachycardias	I45.9
Supraventricular tachycardias	I47.1
Supraventricular tachycardias	I47.9
Supraventricular tachycardias	I48.0
Supraventricular tachycardias	I48.1
Supraventricular tachycardias	I48.2
Supraventricular tachycardias	I48.3
Supraventricular tachycardias	I48.4
Supraventricular tachycardias	I48.9
Supraventricular tachycardias	I49.1
Supraventricular tachycardias	I49.2
Supraventricular tachycardias	I49.4
Supraventricular tachycardias	I49.5
Supraventricular tachycardias	I49.8
Supraventricular tachycardias	I49.9
Supraventricular tachycardias	R00.0
Supraventricular tachycardias	R00.1
Supraventricular tachycardias	R00.2

Supraventricular tachycardias	R00.8
TIA	G45.0
TIA	G45.1
TIA	G45.2
TIA	G45.4
TIA	G45.8
TIA	G45.9
Upper GI haemorrhage	K20.X
Upper GI haemorrhage	K21.0
Upper GI haemorrhage	K21.9
Upper GI haemorrhage	K22.1
Upper GI haemorrhage	K22.6
Upper GI haemorrhage	K25.0
Upper GI haemorrhage	K25.4
Upper GI haemorrhage	K25.6
Upper GI haemorrhage	K26.0
Upper GI haemorrhage	K26.4
Upper GI haemorrhage	K26.6
Upper GI haemorrhage	K27.0
Upper GI haemorrhage	K27.4
Upper GI haemorrhage	K27.6
Upper GI haemorrhage	K28.0
Upper GI haemorrhage	K28.4
Upper GI haemorrhage	K28.6
Upper GI haemorrhage	K92.0
Upper GI haemorrhage	K92.1
Upper GI haemorrhage	K92.2
Upper GI haemorrhage	K29.7
Urinary tract infection	N11.0
Urinary tract infection	N11.1
Urinary tract infection	N11.8
Urinary tract infection	N11.9
Urinary tract infection	N13.6
Urinary tract infection	N30.0
Urinary tract infection	N30.1
Urinary tract infection	N30.2
Urinary tract infection	N30.3
Urinary tract infection	N30.4
Urinary tract infection	N30.8
Urinary tract infection	N30.9
Urinary tract infection	N34.1
Urinary tract infection	N34.2
Urinary tract infection	N34.3
Urinary tract infection	N39.0
Urinary tract infection	N12
