# Supplementary material for: Winter all year round in urgent and emergency care: a large retrospective analysis of routinely collected NHS data across England, 2021–2022
Source: BMC Health Serv Res. 2026 Mar 4;26:499. doi: 10.1186/s12913-026-14253-3 (PMC13067658; doi:10.1186/s12913-026-14253-3)
Supplement: Supplementary file 5 — Supplementary Material 5: Additional results. PDF File containing figures and tables documenting further results not presented in the main text. [file 12913_2026_14253_MOESM5_ESM.pdf]

Additional File 5: Additional results

A5-1. Plots of demographics by site

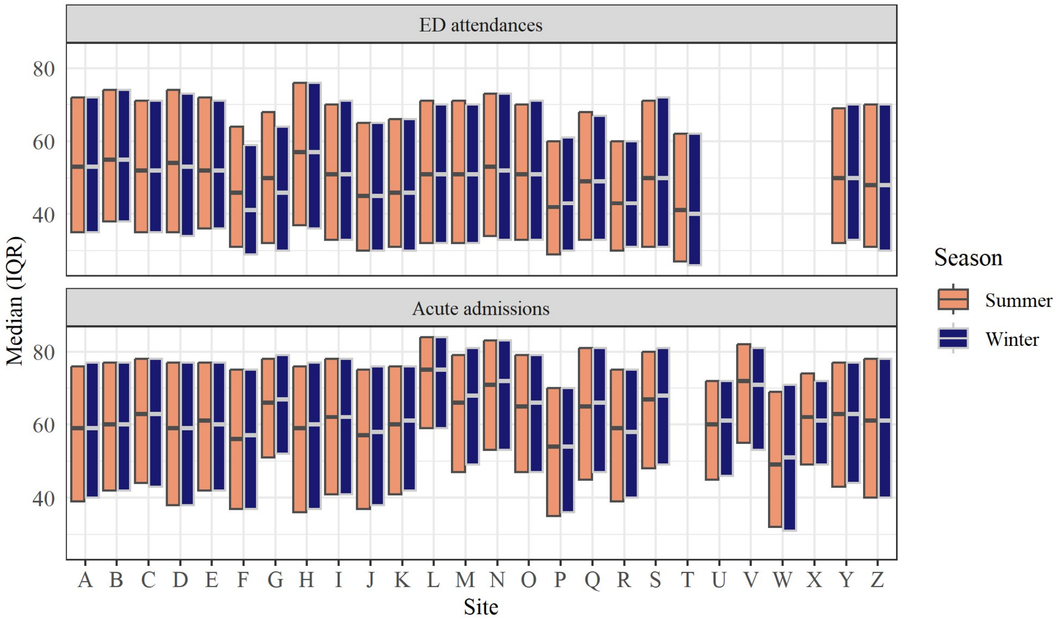

Fig A5-1.1: Median age among ED attendances and acute admissions with interquartile range for each site. Site T did not provide age as a continuous variable for acute admissions.

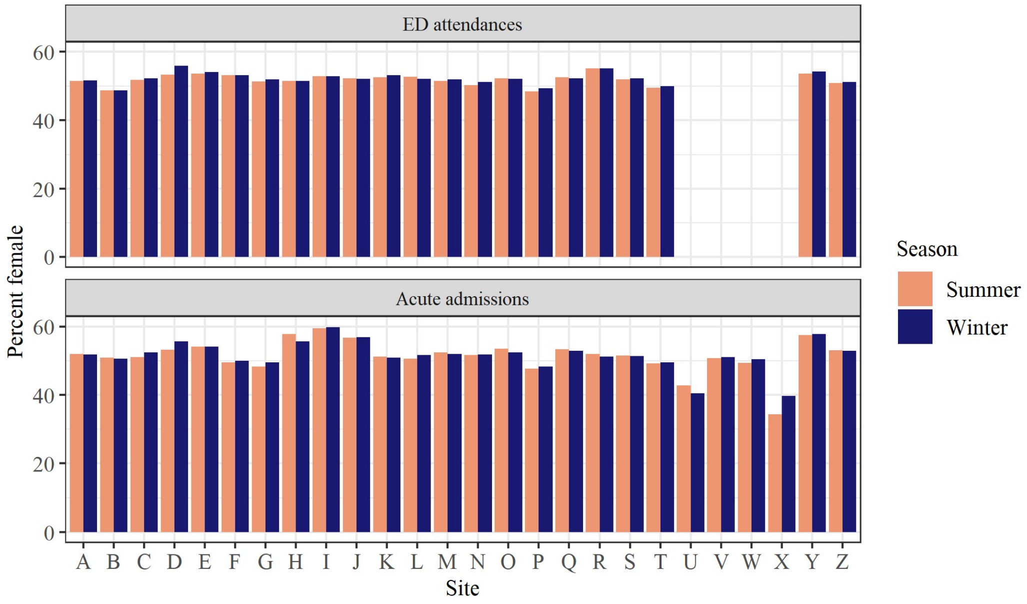

Fig A5-1.2: Percent female patients among ED attendances and acute admissions.

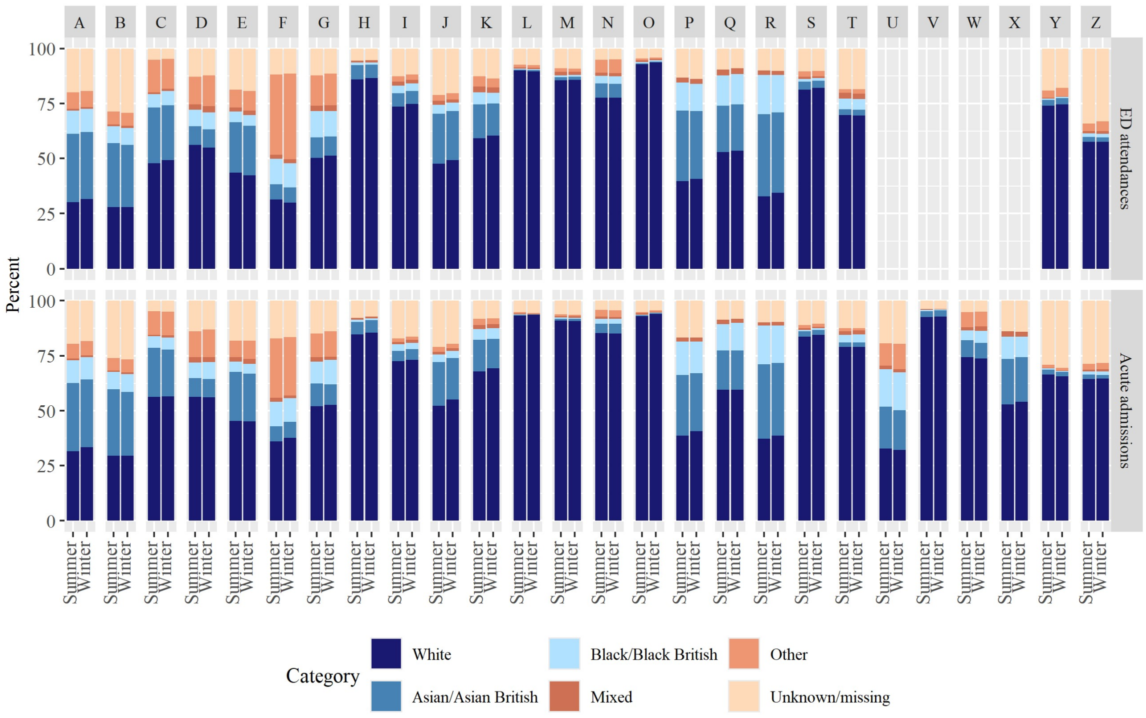

Fig A5-1.3: Distribution of ethnicity among ED attendances and acute admissions.

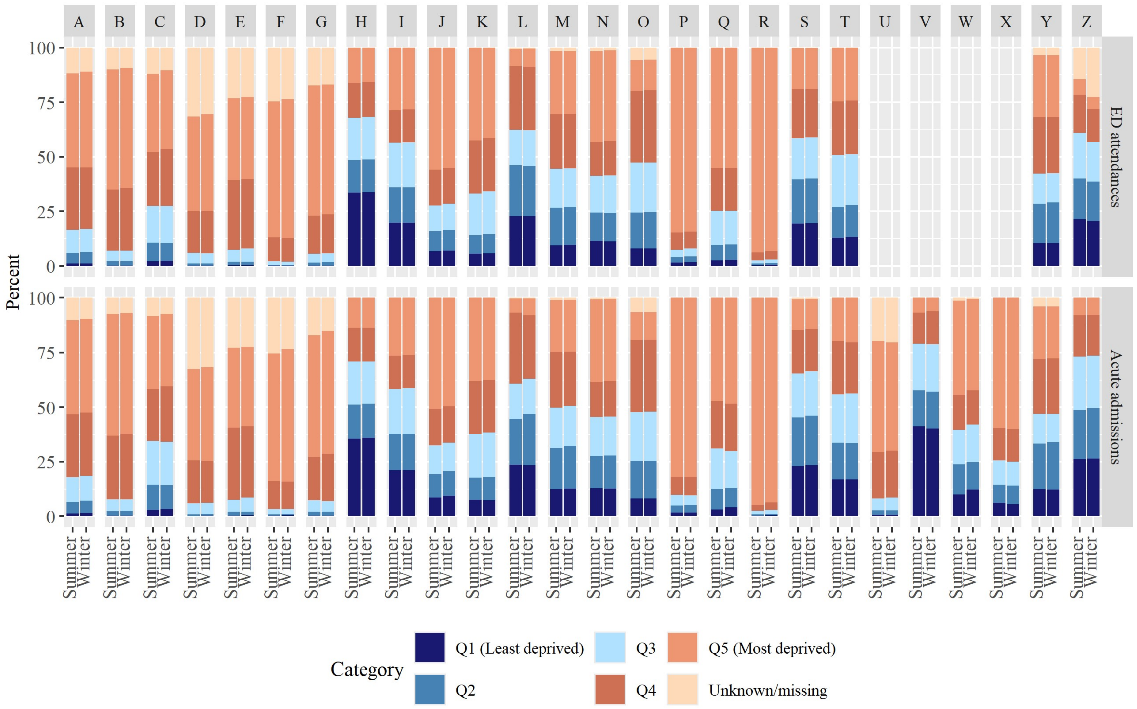

Fig A5-1.4 Distribution of Townsend deprivation quintile among ED attendances and acute admissions.

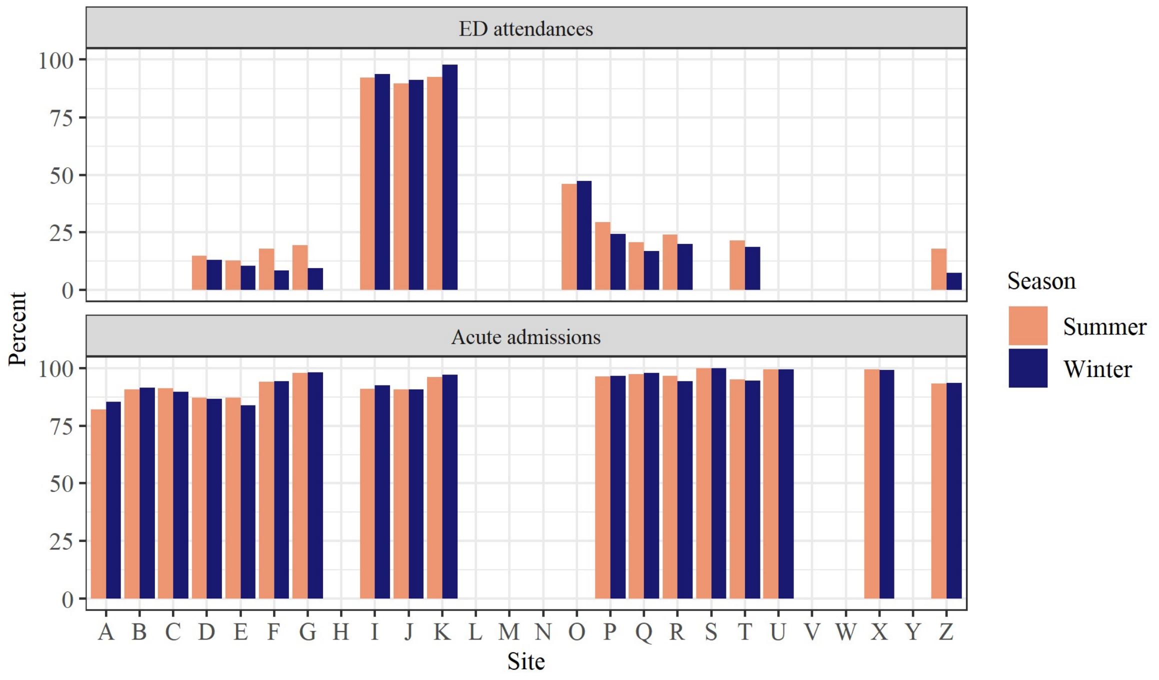

Fig A5-1.5: Percent of ED attendances and acute admissions with recorded comorbidities.

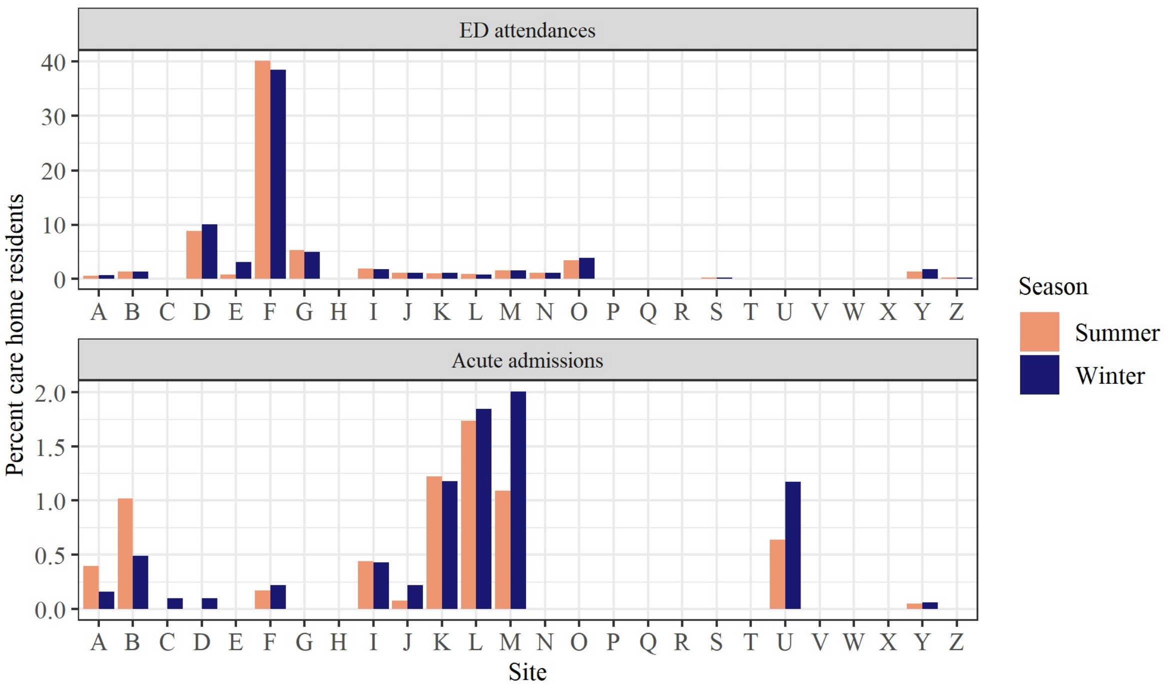

Fig A5-1.6: Percent of ED attendances and acute admissions with recorded care home residence.

A5-2: Plots of key performance indicators by site

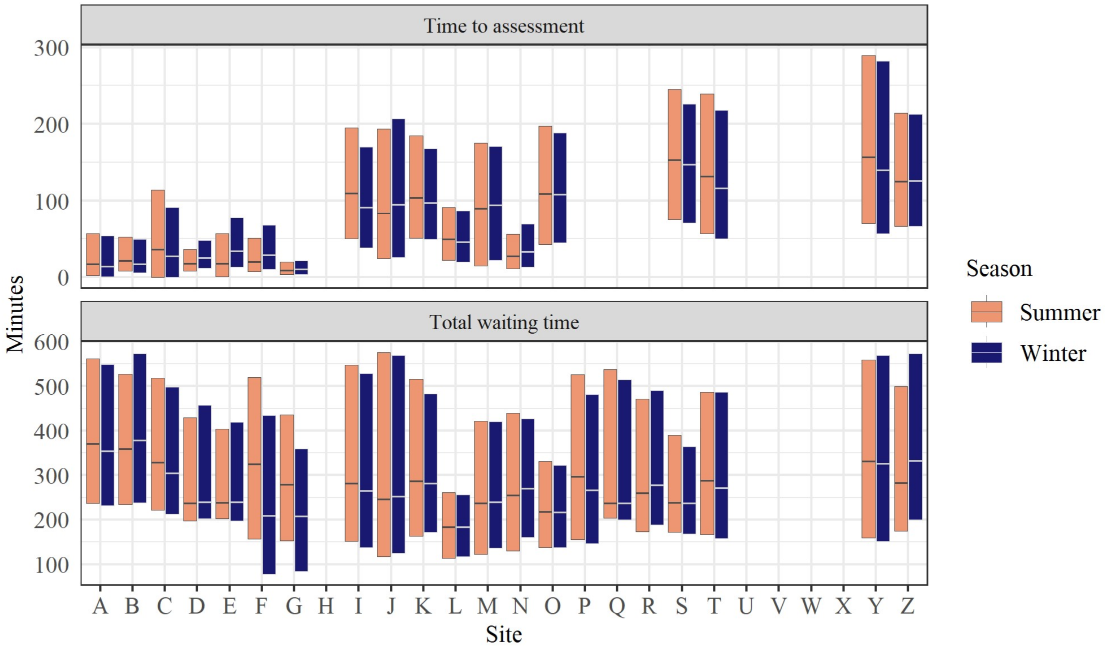

Fig A5-2.1: Median time to assessment and total time in ED (minutes). Boxes show interquartile range for each site.

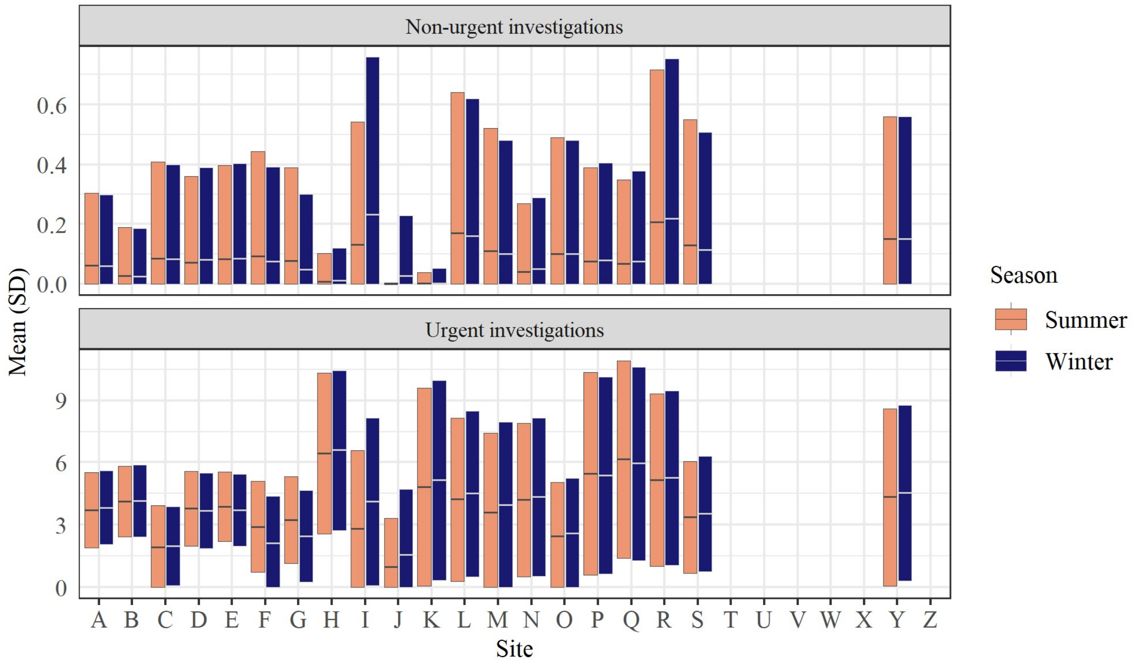

Fig A5-2.2: Mean number of urgent and non-urgent investigations received per ED attendance (± SD, minimum capped at 0). Note that the number of urgent investigations is higher as there are only a small number of possible non-urgent investigations that can be performed.

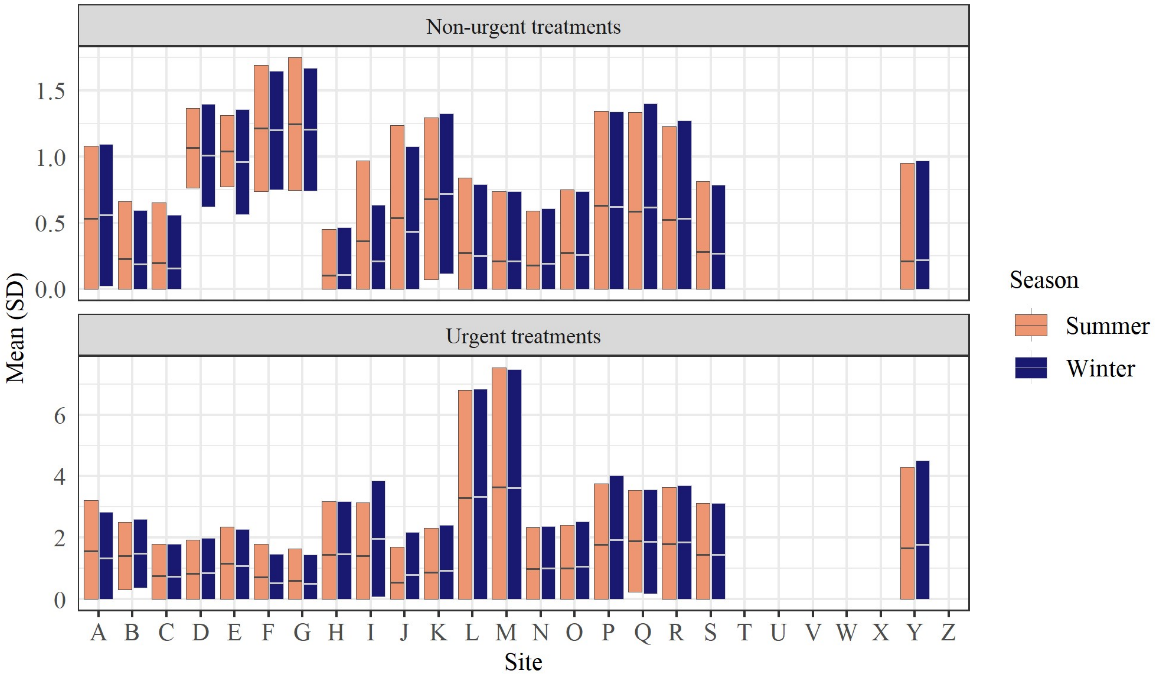

Fig A5-2.3: Mean number of urgent and non-urgent treatments received per ED attendance (± SD, minimum capped at 0). Note that the number of urgent treatments is higher as there are only a small number of possible non-urgent treatments that can be performed.

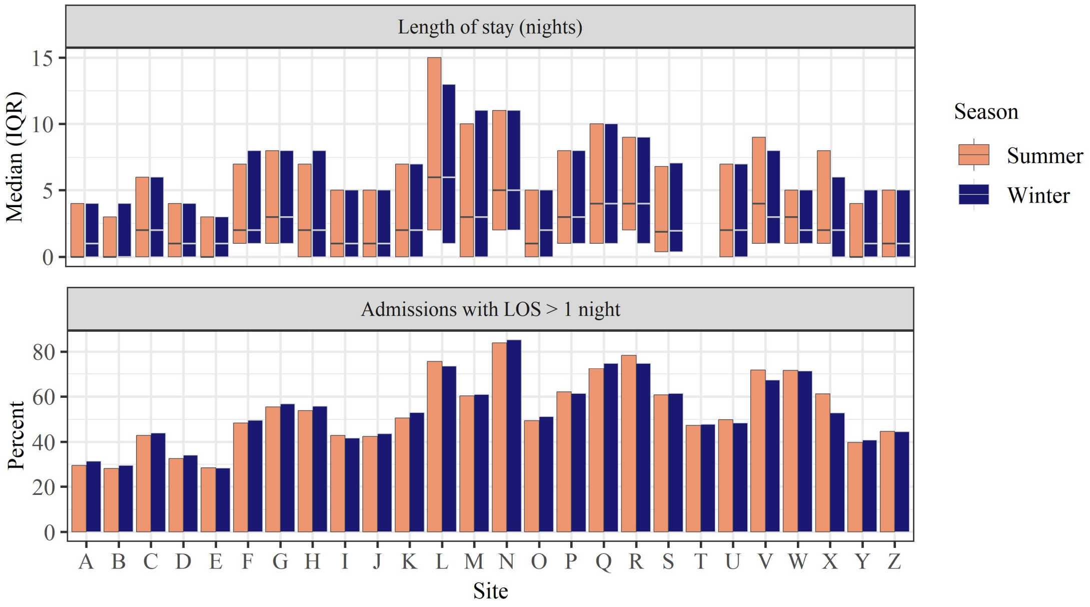

Fig A5-2.4 Median length of stay (nights) with interquartile range, and percent of admissions with length of stay of more than one night

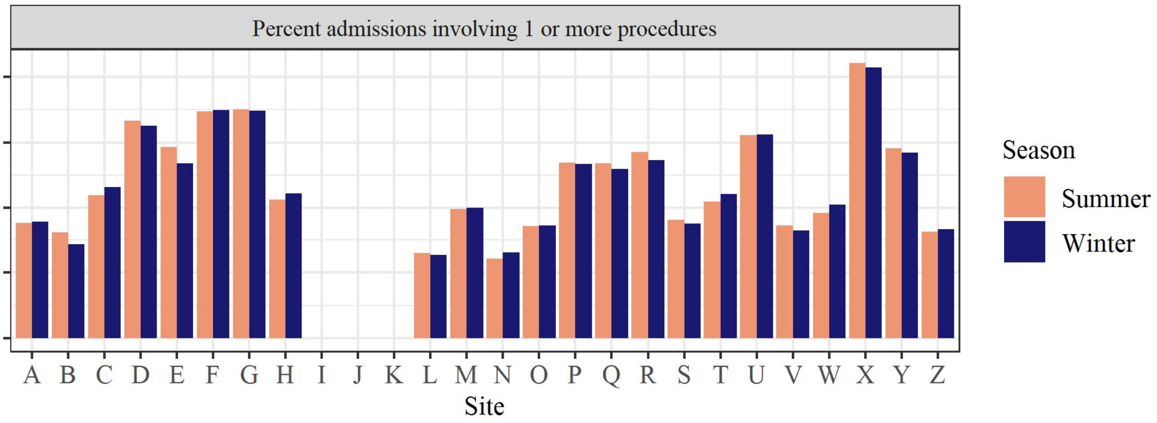

Fig A5-2.5 Percent of admissions at each site involving at least one operative procedure

A5-3: Forest plots for additional key performance indicators

Investigations and treatments in ED

The total number of investigations and treatments (both urgent and non-urgent) were considered together for adjusted logistic regression. These analyses examined the impact of winter on the number of visits involving 2 or more investigations and treatments to understand whether higher-burden patients were seen in winter. Forest plots showing resulting odds ratios are demonstrated in Fig A5-3.1. A small number of sites showed a positive or a negative effect of winter, but most odds ratios were close to 1 (no effect), and no consistent trend in the impact of winter was seen across all sites. Site C was unable to provide reliable estimates for the regression analyses so was excluded.

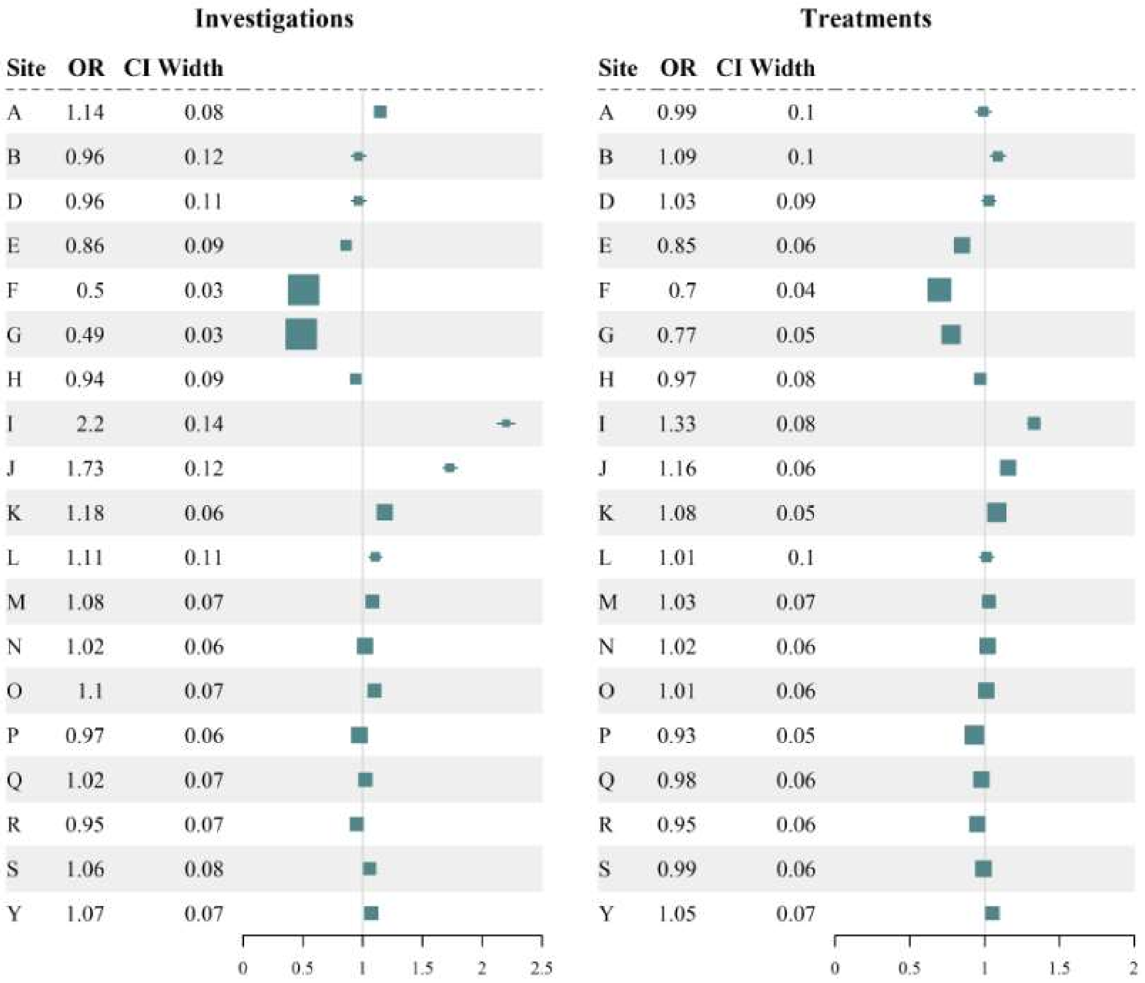

Fig A5-3.1: Forest plots of odds ratio (OR) estimates for the adjusted effect of winter on the number of investigations & treatments received in ED.

A5-4: Summary of clinical characteristics

Pooled results across sites for clinical characteristics of attendances and admissions are shown in tables XX & XX by season and overall. For acute attendances, sites A-G and U were unable to provide time of admission. We did not find notable differences overall between seasons. Source of attendance to and discharge destination from ED showed worse completion rates during winter, but the distribution of recorded responses was similar between seasons. Otherwise all variables were broadly consistent across seasons.

Plots of clinical characteristics of attendances and admissions are shown in Figs A5-5.1 fig to A5-5.9. As with demographics and performance indicators, clinical characteristics of ED attendances and acute admissions showed some variation across sites, but no consistent trends between seasons.

Table A5-4.1 - Pooled percentages for clinical characteristics of ED attendances, with a min-max percentage range across sites.

	October-March		April-September		Overall	
	%	min %-max %	%	min %-max %	%	min %-max %
Arrival mode						
Ambulance	33·83	21·47 - 51·21	33·13	19·85 - 49·36	33·48	20·62 - 50·27
Walk-in/Other	65·67	48·79 - 76·50	66·43	50·64 - 72·98	66·05	49·73, 73·47
Unknown/ missing	0·50	0·00 - 8·77	0·45	0·00 - 7·17	0·47	0·00 - 7·93
Attendance source						
Personal	53·52	0·00 - 84·95	52·14	0·00 - 88·20	52·83	0·00 - 86·57
Primary care	10·60	2·50 - 20·48	9·56	1·83 - 21·30	10·08	2·47 - 20·90
Emergency services	17·66	0·10 - 36·42	16·16	0·10 - 39·35	16·91	0·10 - 37·48
Hospital	7·76	0·23 - 54·36	7·61	0·30 - 52·07	7·69	0·30 - 53·32
Community	0·22	0·00 - 0·74	0·23	0·00 - 0·92	0·23	0·00 - 0·81
Unknown/ missing	10·24	0·00 - 85·28	14·30	0·00 - 87·65	12·27	0·00 - 86·47
Time of attendance						
In-hours	44·07	37·35 - 47·41	42·91	36·81 - 46·63	43·49	37·09 - 47·01
Out-of-hours	55·93	52·59 - 62·65	57·09	53·37 - 63·19	56·51	52·99 - 62·91
Seasonal diagnosis						
Chronic disease exacerbation	1·58	0·85 - 3·01	1·50	0·63 - 2·52	1·54	0·78 - 2·76
Respiratory infection	3·99	1·66 - 5·74	3·63	1·96 - 4·75	3·81	1·91 - 5·25
None	93·21	79·35 - 97·38	93·42	79·83 - 97·17	93·32	79·62 - 97·23
Unknown/ missing	1·22	0·00 - 14·42	1·45	0·00 - 15·12	1·34	0·00 - 14·81
Attendance acuity						
Immediate	4·11	0·14 - 20·70	4·26	0·10 - 25·08	4·18	0·12 - 22·90
Very urgent	7·74	0·00 - 29·20	8·12	0·00 - 31·26	7·93	0·00 - 30·16
Urgent	41·40	1·07 - 79·96	41·00	0·52 - 79·67	41·20	0·79 - 79·81
Standard	39·57	0·00 - 77·19	39·83	0·00 - 74·12	39·70	0·00 - 75·63
Low acuity	2·81	0·00 - 19·89	2·90	0·00 - 18·69	2·86	0·00 - 19·28
Unknown/ missing	4·37	0·00 - 27·87	3·90	0·00 - 26·13	4·13	0·00 - 26·98
Chief complaint						
Airway/ breathing	8·39	6·52 - 10·49	7·06	5·43 - 8·15	7·72	5·97 - 9·16
Circulation/chest	15·42	11·40 - 21·11	14·72	11·56 - 20·09	15·07	11·86 - 20·59
Drug and alcohol	1·36	0·49 - 3·62	1·44	0·49 - 3·21	1·40	0·49 - 3·41
Environmental	0·59	0·11 - 3·14	0·60	0·07 - 3·27	0·60	0·09 - 3·20
Eye	1·11	0·23 - 2·35	1·15	0·18 - 2·57	1·13	0·23 - 2·46
Gastrointestinal	11·56	8·70 - 15·52	12·19	9·59 - 16·12	11·88	9·25 - 15·62
General	7·27	2·53 - 13·46	7·43	2·83 - 15·11	7·35	2·68 - 14·30
Genitourinary	3·76	2·02 - 6·19	3·87	2·48 - 7·32	3·82	2·26 - 6·67
Head and neck	3·65	1·12 - 7·27	3·57	1·11 - 5·59	3·61	1·12 - 6·56

Injury	16·42	5·67 - 24·53	17·05	4·64 - 25·67	16·73	5·72 - 25·11
Musculoskeletal	6·48	3·84 - 10·48	6·93	4·08 - 11·03	6·70	3·96 - 10·76
Neurological	8·98	6·08 - 17·39	9·30	6·19 - 17·61	9·14	6·13 - 17·50
ObGyn	2·09	0·87 - 5·88	2·05	0·89 - 6·00	2·07	0·91 - 5·83
Psychosocial/ behaviour change	1·73	0·70 - 3·29	2·08	0·68 - 3·64	1·91	0·73 - 3·46
Skin	4·76	1·11 - 8·92	5·28	1·57 - 8·63	5·02	1·47 - 8·01
Unknown/ missing	6·42	0·22 - 17·44	5·27	0·00 - 16·24	5·84	0·11 - 16·53
Discharge destination						
Discharged	63·07	28·62 - 81·71	63·99	39·75 - 82·42	63·53	38·58 - 82·08
Ambulatory/ short stay	4·58	0·00 - 23·40	4·98	0·00 - 34·41	4·78	0·00 - 22·99
Admitted	24·79	4·76 - 42·79	25·82	8·21 - 41·97	25·30	6·34 - 42·39
Transfer	2·08	0·23 - 14·05	2·08	0·28 - 15·20	2·08	0·26 - 14·64
Died	0·15	0·00 - 0·31	0·14	0·00 - 0·28	0·14	0·00 - 0·29
Unknown/ missing	5·33	0·00 - 58·04	2·99	0·00 - 25·65	4·16	0·00 - 34·00

Table A5-4.2 - Pooled percentages for clinical characteristics of acute admissions, with a min-max percentage range across sites.

	October-March		April-September		Overall	
	%	min %-max %	%	min %-max %	%	min %-max %
Admission source						
Residence	85·51	10·69 - 99·66	86·26	11·22 - 99·81	85·89	10·96 - 99·69
Care home	0·31	0·00 - 2·01	0·28	0·00 - 1·74	0·30	0·00 - 1·79
Medical care	13·86	0·09 - 89·31	13·15	0·00 - 88·78	13·5	0·04 - 89·04
Penal	0·02	0·00 - 0·33	0·02	0·00 - 0·28	0·02	0·00 - 0·31
Unknown/missing	0·29	0·00 - 6·12	0·29	0·00 - 6·53	0·29	0·00 - 6·33
Discharge destination						
Residence	91·50	80·95 - 96·75	91·90	79·86 - 97·69	91·7	80·4 - 97·21
Care home	1·76	0·00 - 8·24	1·82	0·00 - 9·51	1·79	0·00 - 8·88
Medical care	2·25	0·47 - 10·73	2·24	0·39 - 12·55	2·24	0·43 - 11·56
Penal	0·05	0·00 - 0·28	0·03	0·00 - 0·26	0·04	0·00 - 0·26
Died	3·73	1·02 - 8·65	3·32	1·08 - 7·67	3·52	1·13 - 8·16
Unknown/missing	0·70	0·00 - 13·9	0·69	0·00 - 15·49	0·70	0·00 - 14·66
Seasonal diagnosis						
Chronic disease exacerbation	2·98	0·00 - 5·55	2·69	0·00 - 4·80	2·83	0·00 - 5·17
Respiratory infection	6·85	0·89 - 17·37	5·86	0·43 - 16·45	6·35	0·68 - 16·91
None	90·17	77·08 - 98·39	91·45	78·75 - 99·15	90·82	77·92 - 98·74
Time of admission						
In-hours	43·04	28·06 - 54·69	43·67	26·63 - 57·40	43·36	27·32 - 56·05
Out-of-hours	56·95	45·31 - 71·94	56·32	42·60 - 73·37	56·63	43·95 - 72·68
Unknown/missing	0·01	0·00 - 0·24	0·01	0·00 - 0·20	0·01	0·00 - 0·22

A5-5: Plots of clinical characteristics by site

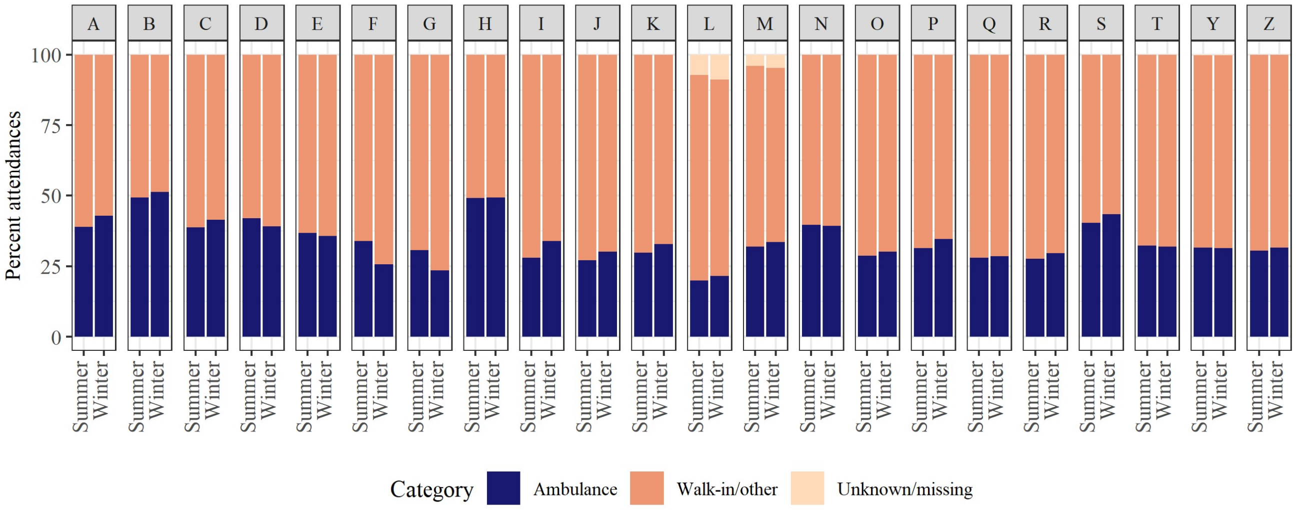

Fig A5-5.1: Percentage of ED attendances arriving by ambulance or other means.

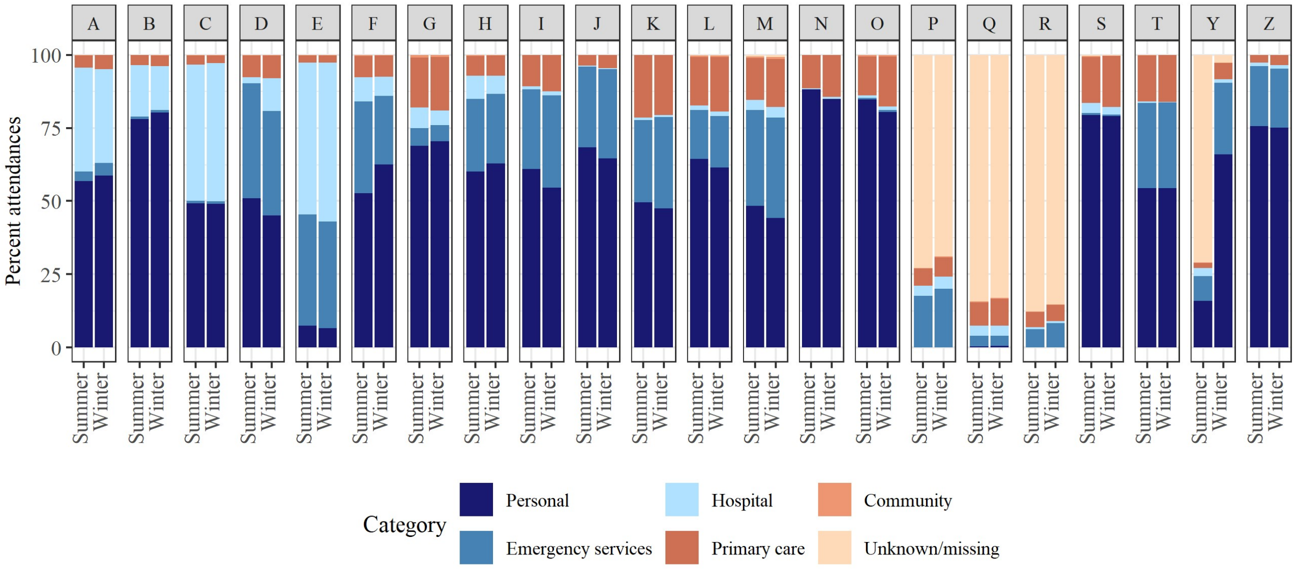

Fig A5-5.2: Distribution of source of referral for ED attendances

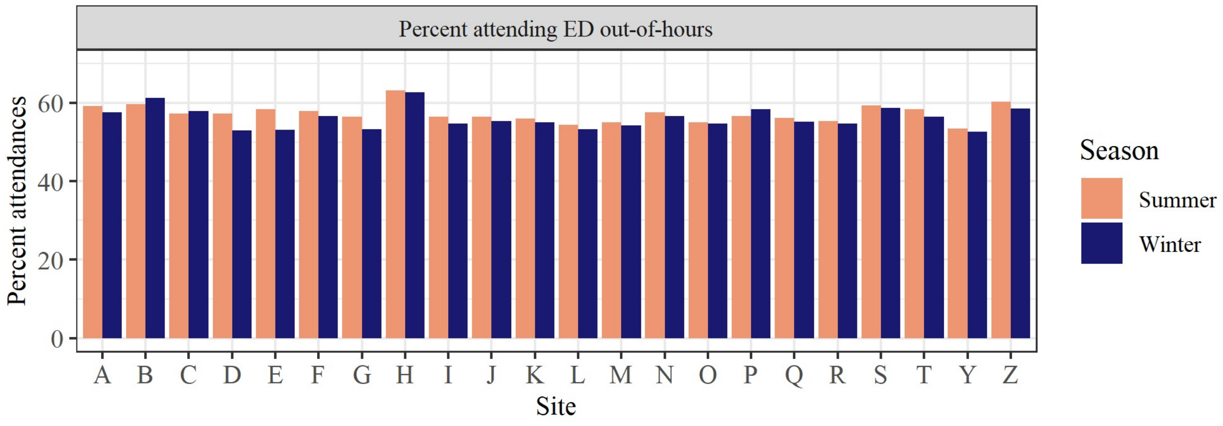

Fig A5-5.3: Percentage of ED attendances occurring 'out-of-hours' (Outside the working hours of 8am-6pm Monday-Friday)

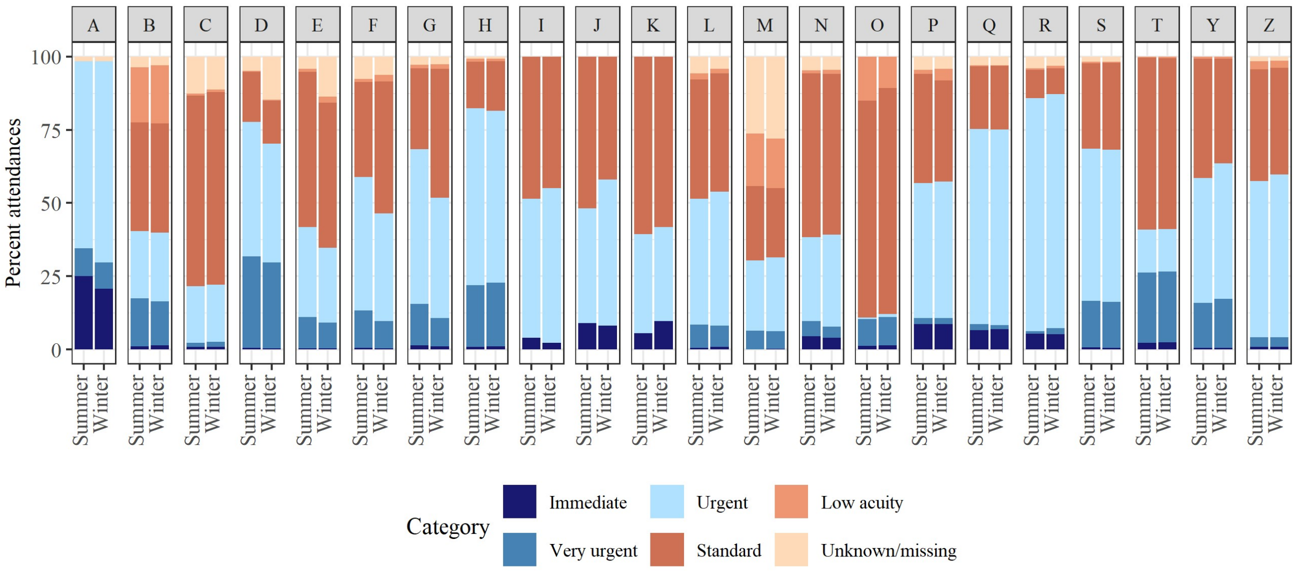

Fig A5-5.4: Distribution of acuity rating for ED attendances

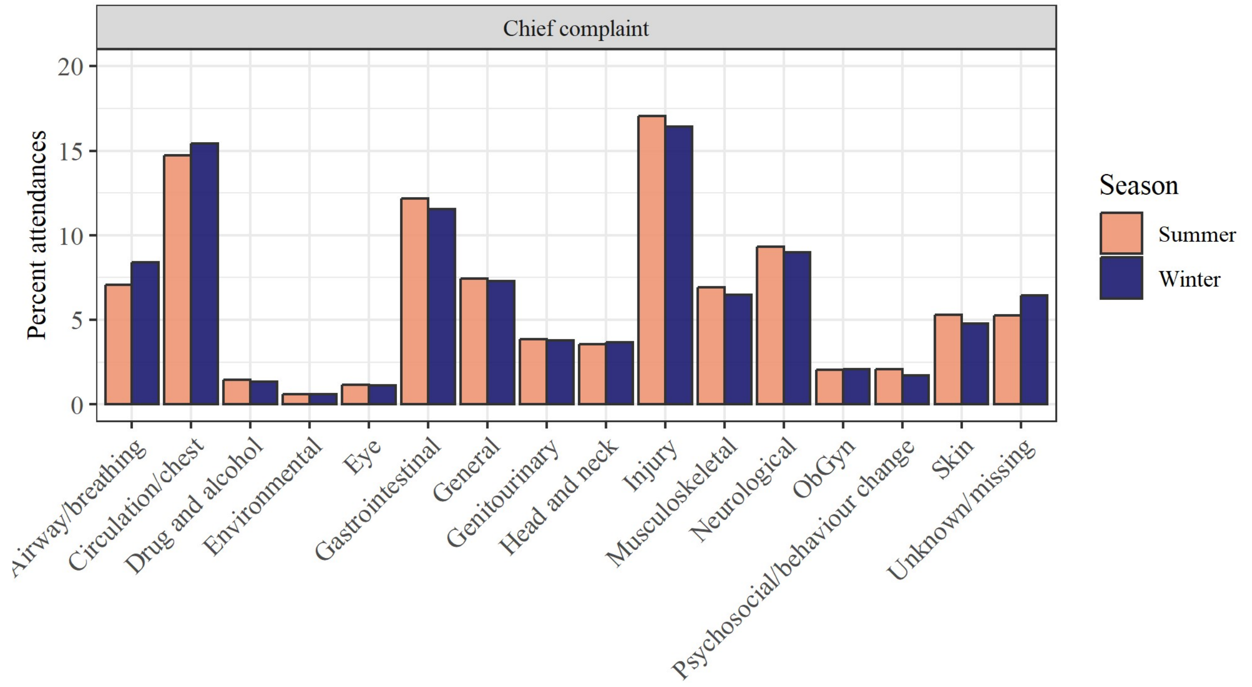

Fig A5-5.5: Chief complaint registered for ED attendances. Aggregated across sites for ease of visualisation.

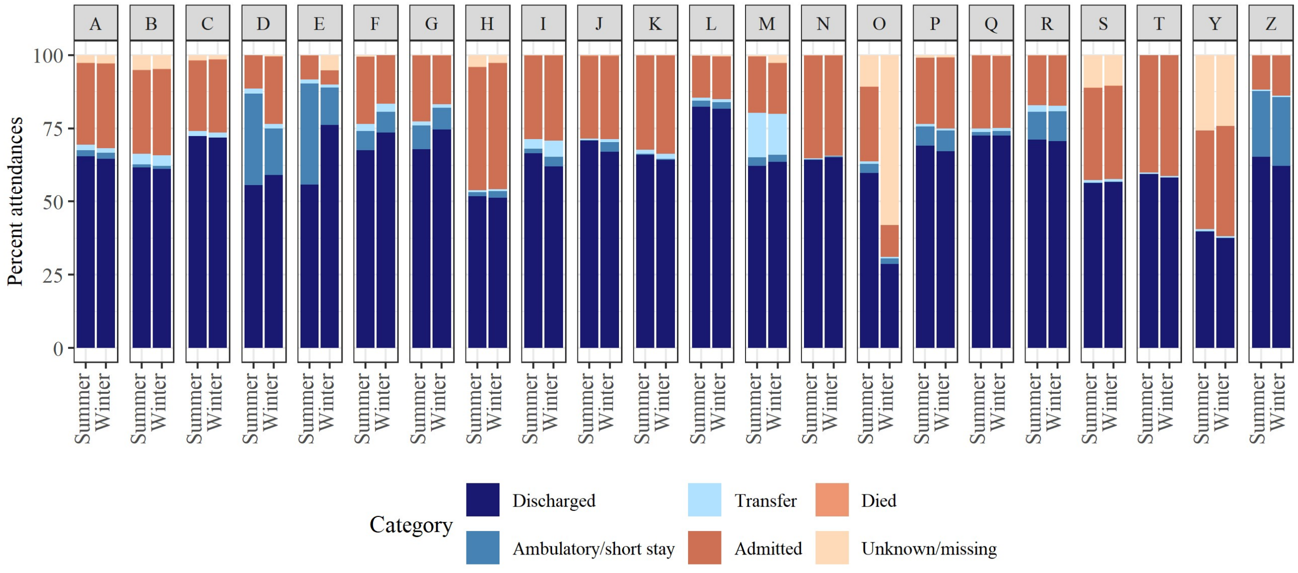

Fig A5-5.6: Discharge destination of ED attendances

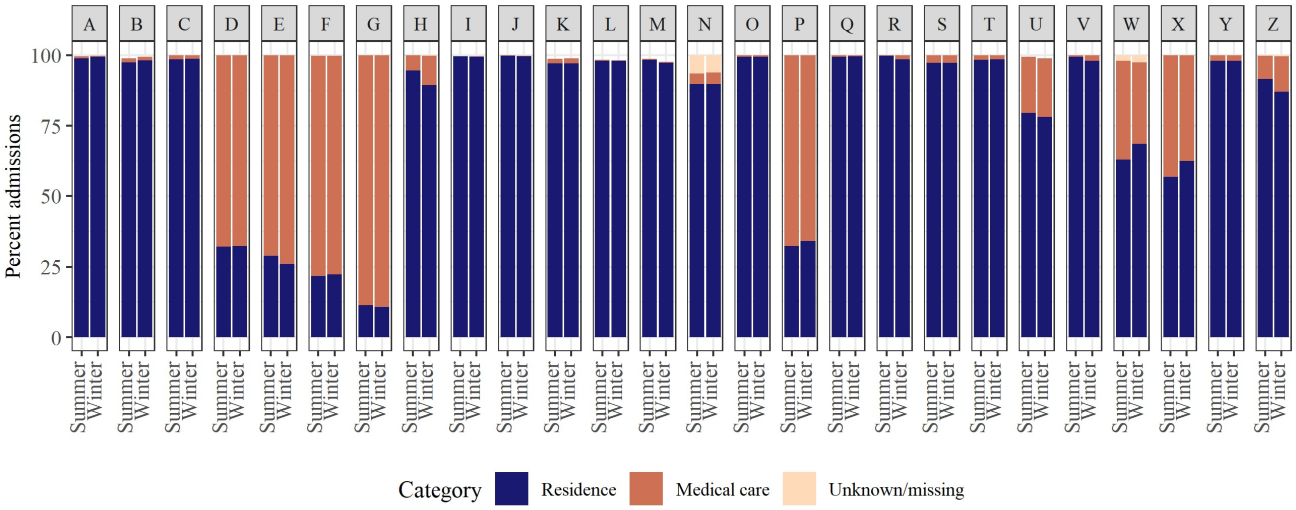

Fig A5-5.7: Admission source for acute admissions (care home and penal sources of admission omitted from plot due to small numbers)

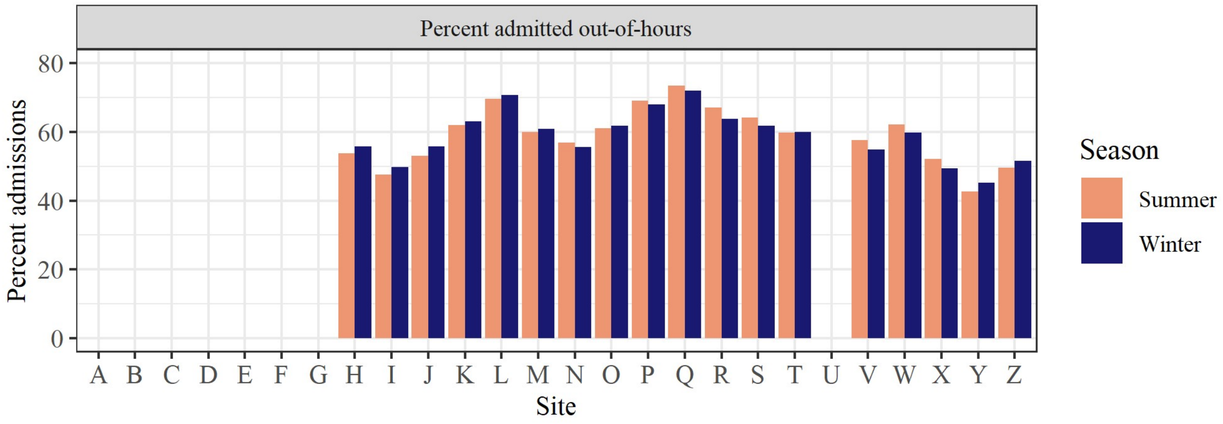

Fig A5-5.8: Percentage of acute admissions occurring 'out-of-hours' (Outside the working hours of 8am-6pm Monday-Friday). Time of admission was missing for sites A-G and U.

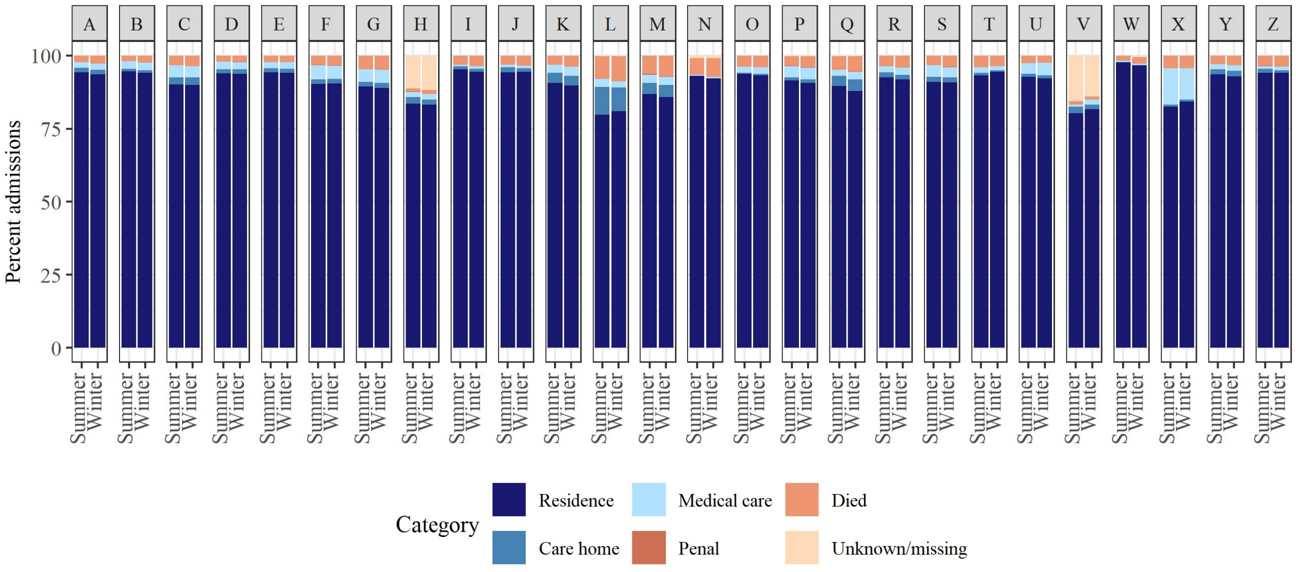

Fig A5-5.9: Discharge destination of acute admissions
